# Breast cancer patient-derived microtumors resemble tumor heterogeneity and enable protein-based stratification and functional validation of individualized drug treatment

**DOI:** 10.1186/s13046-023-02782-2

**Published:** 2023-08-18

**Authors:** Nicole Anderle, Felix Schäfer-Ruoff, Annette Staebler, Nicolas Kersten, André Koch, Cansu Önder, Anna-Lena Keller, Simone Liebscher, Andreas Hartkopf, Markus Hahn, Markus Templin, Sara Y. Brucker, Katja Schenke-Layland, Christian Schmees

**Affiliations:** 1 NMI Natural and Medical Sciences Institute at the University of Tuebingen, 72770 Reutlingen, Germany; 2 Institute of Pathology and Neuropathology, Eberhard Karls University Tuebingen, 72076 Tuebingen, Germany; 3 Interfaculty Institute for Bioinformatics and Medical Informatics (IBMI), Eberhard Karls University Tuebingen, Tuebingen, 72076 Germany; 4 FZI Research Center for Information Technology, 76131 Karlsruhe, Germany; 5 Department of Women’s Health, University Women’s Hospital, Eberhard Karls University Tuebingen, 72076 Tuebingen, Germany; 6 Institute of Biomedical Engineering, Department for Medical Technologies and Regenerative Medicine, Eberhard Karls University Tuebingen, 72076 Tuebingen, Germany; 7 Department of Gynecology and Obstetrics, University Hospital of Ulm, 89081 Ulm, Germany; 8 Cluster of Excellence iFIT (EXC2180) ”Image-Guided and Functionally Instructed Tumor Therapies”, Eberhard Karls University Tuebingen, 72076 Tuebingen, Germany

**Keywords:** Preclinical tumor model, Tumor heterogeneity, Therapy resistance, Therapy sensitivity, Protein profiling, Breast cancer, Anti-cancer drug efficacy

## Abstract

Despite tremendous progress in deciphering breast cancer at the genomic level, the pronounced intra- and intertumoral heterogeneity remains a major obstacle to the advancement of novel and more effective treatment approaches. Frequent treatment failure and the development of treatment resistance highlight the need for patient-derived tumor models that reflect the individual tumors of breast cancer patients and allow a comprehensive analyses and parallel functional validation of individualized and therapeutically targetable vulnerabilities in protein signal transduction pathways. Here, we introduce the generation and application of breast cancer patient-derived 3D microtumors (BC-PDMs). Residual fresh tumor tissue specimens were collected from *n* = 102 patients diagnosed with breast cancer and subjected to BC-PDM isolation. BC-PDMs retained histopathological characteristics, and extracellular matrix (ECM) components together with key protein signaling pathway signatures of the corresponding primary tumor tissue. Accordingly, BC-PDMs reflect the inter- and intratumoral heterogeneity of breast cancer and its key signal transduction properties. DigiWest®-based protein expression profiling of identified treatment responder and non-responder BC-PDMs enabled the identification of potential resistance and sensitivity markers of individual drug treatments, including markers previously associated with treatment response and yet undescribed proteins. The combination of individualized drug testing with comprehensive protein profiling analyses of BC-PDMs may provide a valuable complement for personalized treatment stratification and response prediction for breast cancer.

**Supplementary Information:**

The online version contains supplementary material available at 10.1186/s13046-023-02782-2.

## Background

According to the SEER (The Surveillance, Epidemiology, and End Results—Program) database, breast cancer (BC) remains the most common cancer in women. Despite a 5-year survival rate of 90% (all cancer stages), BC is the 2^nd^ leading cause of cancer death in women. Since 1989, BC mortality rates have been reduced by 43%, primarily through early detection by mammography, improved local treatment, and increasingly effective systemic adjuvant therapies in early stages of cancer [1]. Based on the genetic, morphologic, and clinical intertumoral heterogeneity, BC is classified into different subtypes. The WHO distinguishes 19 different histological subtypes including invasive BC, which infiltrate the stroma and surrounding breast tissue, and non-invasive, in-situ carcinomas, which are the preinvasive counterparts. If they arise in the mammary ducts, they are referred to as invasive ductal carcinomas (IDC) or ductal carcinoma in-situ (DCIS). Whereas invasive lobular carcinomas (ILC) and lobular carcinomas in-situ (LCIS) arise from the lobules of the mammary glands [2]. The most common invasive subtype is IDC of no special type (NST) showing no distinct architectural features [3]. IDC subtypes with defined, distinctive architectural features are less common. Global gene expression analyses have further classified BC into four molecular subtypes with distinct gene expression patterns: the hormone receptor-related luminal A and luminal B tumors versus the hormone receptor-negative, HER2-enriched and basal-like tumors [4–6]. These reflect different phenotypes, disease prognosis, treatment paradigms and responses to therapies [7–11]. In clinical practice, BC stratification is performed by the immunohistochemical determination of routine pathologic markers such as estrogen receptor α (ERα), progesterone receptor (PgR) and human epidermal growth factor receptor 2 (HER2), and by semiquantitative evaluation of Ki-67. In this regard, BC is pathologically classified as ERα/PR-positive, HER2-positive or as triple-negative breast cancer (TNBC), which lack the expression of these receptors and can themselves be considered a very heterogeneous group of cancers [12, 13]. Besides this intertumoral heterogeneity, enormous diversity of tumor cell profiles is also observed within the same tumor, termed intratumoral heterogeneity [14]. Alterations in genome, epigenome/transcriptome, and proteome, in invasive capacity, proliferation, stemness, cell plasticity but also the extrinsic interplay with the tumor microenvironment [15] contribute to the heterogeneity of individual tumor cell subpopulations. This leads to diverse disease manifestations in individual patients and failure of systematic treatment [16]. With regard to the TME, we are only at the beginning of our understanding of its interaction with the tumor and how it influences the response to therapy [17, 18]. Apparently, different TME gene expression patterns alter BC phenotypes [19, 20]. Despite the success of genomic expression analysis in classifying BC according to different gene signatures or revealing gene alterations, a comprehensive understanding of treatment failures due to extensive tumor heterogeneity is still lacking [21, 22]. Therefore, more effective therapies need to be developed and the mechanisms of resistance better understood. In particular, a personalized treatment approach based on functional analysis of protein expression data could help to improve treatment efficacy and patient outcome.

Here, we demonstrate the applicability of patient-derived microtumors (PDM) isolated from residual fresh mammary carcinoma tissue samples as an ex vivo 3D breast cancer model that not only consists of tumor cells but also of TME and extracellular matrix (ECM) components of the corresponding patient tumor. We successfully generated microtumor samples of different BC subtypes with histopathological features and ECM components corresponding to those of the original primary tumor tissue. Protein profiling of BC-PDMs by DigiWest® revealed heterogeneous signaling pathway activity similar to the patient´s tumor and reflected the intertumoral heterogeneity of BC. We combined functional drug testing with signaling pathway analyses in BC-PDMs to evaluate therapy responses and identified markers of treatment sensitivity/resistance.

## Materials and methods

### Human specimen

Non-processed human breast tumor samples were collected after surgery and completion of pathological examination from patients with primary breast cancer as part of the publicly funded PRIMO project (Personalized medicine for tailored cancer therapies). Written informed consent was obtained from all participants prior to surgery. The research project was approved by the ethics commission at the Medical Faculty Tuebingen (project number #788/2018BO2). Clinical patient data for the above-mentioned samples were submitted in pseudonymized form. A total of *n* = 102 samples were obtained from consenting participants, who underwent surgery at Center for Women’s Health, University Hospital Tuebingen. Inclusion criteria were individuals > 18 years of age who had given informed consent to participate in the project, with unilateral invasive primary and recurrent breast carcinomas regardless of ER-/PgR- and HER2-status, tumor size, nodal-status and grading. Enrolled patients did not receive neoadjuvant treatment. Patients with distant metastatic disease were excluded.

### Generation of patient-derived microtumors from residual fresh breast tumor tissue

Fresh dissected breast tumor tissues were transported within DMEM/F12 culture media (Gibco) and subsequently processed as previously described [23]. The isolation of patient-derived microtumors was adapted from Kondo et al. [24]. Briefly, tumors were washed in HBSS (Gibco), fragmented with forceps, and digested with Liberase DH (Roche) for 2 h at 37 °C. The digested tissue was filtered through a 500 µm stainless steel mesh (VWR) followed by a 40 µm cell strainer (Corning). Tumor fragments retained by the cell strainer were washed in HBSS and cultured in suspension in StemPro® hESC SFM (Gibco) supplemented with 8 ng/ml FGF-basic (Gibco), 0.1 mM β-mercaptoethanol (Gibco), 1.8% BSA (Gibco) and 100 µg/ml Primocin (Invivogen) in a cell-repellent culture dish (60 × 15 mm) (Corning). The single-cell filtrate was used for the expansion of tumor-infiltrating lymphocytes in Advanced RPMI 1640 (GIBCO) supplemented with 2 mM glutamine (Gibco), 1% MEM vitamins (Gibco), 5% human serum (SigmaAldrich) and 100 µg/ml primocin (Invivogen). IL-2 (100 U/ml), IL-7 (10 U/ml) and IL-15 (23.8 U/ml) (Peprotech) were freshly added to the culture media. CD3/CD28 Dynabeads (Milteny Biotech) were added for expansion.

### Viability measurement of BC-PDMs using Calcein-AM live cell and SYTOX™ orange dead cell stain

Viability of BC-PDMs was assessed by live/dead-cell staining using 6.6 µM Calcein-AM™ (Invitrogen) live cell stain and 5 µM SYTOX™ Orange nucleic acid dead cell stain (Invitrogen). To visualize nuclei 1 μg/mL of Hoechst 33258 (Invitrogen) was added. BC-PDMs were directly picked from the suspension culture and resuspended in staining solution consisting of DMEM/F12 phenol-red free media (Gibco) supplemented with StemPro® hESC supplement (Gibco), 8 ng/ml FGF-basic (STEMCELL Technologies), 0.1 mM 2-mercapto-ethanol (Gibco), 1.8% BSA (Gibco) and 100 µg/ml primocin (Invivogen). After 30 min of incubation, z-stack images were taken using the Zeiss CellObserver Z1 (Carl Zeiss). Maximum intensity projections of the 3D z-stacks were generating using the ZEN software (Version 2.6). Imaris software (version 8.0) was used to create 3D surface masks for viable and dead cells in the FITC and TRITC channel. For each surface mask, the fluorescent intensity sums and the volume was measured. Fluorescent intensities were normalized to the total (BC-PDMs) volume (µm^3^).

### Histology and immunohistochemistry

For histology BC-PDMs were fixed for 1 h in 4% Roti® Histofix (Carl Roth) at RT and incubated for 5 min in Harris Hematoxylin (Leica Biosystems), shortly washed in dH_2_O and dehydrated in an ethanol series (2 × 50% ethanol, 2 × 70% ethanol, each for 15 min). Using Tissue-Tek® Cryomolds® (Sakura), BC-PDMs were embedded in Richard-Allan Scientific™ HistoGel™ (Thermo Fisher Scientific). Tissue processing was performed using the HistoCore PEARL (Leica Biosystems). After processing, BC-PDMs histogel-blocks were paraffin-embedded for sectioning. Three micrometer sections of FFPE BC-PDMs samples were cut. In contrast, corresponding PTT were snap frozen on dry ice and cut as cryosections (5–7 µm). PTT cryosections were immersed in ice-cold 4% Roti® Histofix (Carl Roth) for 10 min at 2–4 °C and washed afterwards 3 × with PBS. Hematoxylin and eosin (H&E) as well as Movat-pentachrome staining was performed on BC-PDMs FFPE and PTT cryosections. Immunohistochemical staining of BC-PDMs was performed using the Autostainer Link 48 (Agilent) in combination with the Dako PT Link (Agilent) for antigen-retrieval according to the manufacturer’s recommendations. Detailed information of the used antibodies is listed below (Table 1). Stained FFPE/cryosections were imaged with Axio Scan Z1. All primary antibodies were validated in normal, healthy tissues as well as in FFPE and cryosections. DAB and collagen staining (Movat-pentachrome staining) was semi-quantified using ImageJ Fiji software. The color deconvolution plugin was used to separate stains using Ruifrok and Johnston's method for DAB stains [25], and manual deconvolution for collagen stain. The percentage of area positive for DAB/collagen was determined. Percent area fraction was measured as the percentage of pixels in the image or selection to which thresholds were applied. The certified pathologist was blinded for evaluation of microtumor H&E stainings.

**Table 1:** Antibodies for IHC staining

Antibody	Manufacturer	Product No	Additional reagents	Usage
rabbit anti-human ERalpha	Abcam	ab16660	Rb Linker Enhancer	1:30
rabbit anti-human HER2/ErbB2	Cell Signaling Technology	4290	Rb Linker Enhancer	1:80
mouse anti-human PgR	Dako	IR068	Ms Linker	R.T.U
rabbit anti-human cytokeratin 5	Abcam	ab64081	Rb Linker Enhancer	1:200
rabbit anti-human cytokeratin 6	Abcam	ab93279	Rb Linker Enhancer	1:50
mouse anti-human cytokeratin 18	Dako	IR618	Ms Linker Enhancer	R.T.U
rabbit anti-human FAPalpha	BioRad	AHP1322	Rb Linker	1:50
rabbit anti-human CD163	Abcam	ab182422	Rb Linker	1:200
mouse anti-human PD-L1	Dako	22C3	Ms Linker Enhancer	1:50
mouse anti-human CD8	Dako	IR623	-	R.T.U

### Multiplex protein profiling via DigiWest®

DigiWest® was performed as described previously [26]. Western blot was carried out using the NuPAGE system (Life Technologies) with a 4–12% Bis–Tris gel and PVDF membranes. Membranes were washed with PBST and proteins were biotinylated by adding 50 µM NHS-PEG12-Biotin in PBST for 1 h. The membranes were washed with PBST and dried overnight. Each protein (Western-Blot) lane was cut into 96 strips of 0.5 mm each. Western Blot-strips were sorted by molecular weight into a 96-well plate (Greiner Bio-One). Proteins were eluted using a 10 µl of elution buffer (8 M Urea, 1% Triton-X100 in 100 mM Tris–HCl pH 9.5). Proteins of each 96-well representing a distinct molecular weight fraction were coupled overnight to Neutravidin-coated MagPlex beads (Luminex) of a distinct color ID. Non-bound binding sites were blocked with 500 µM deactivated NHS-PEG12-Biotin for 1 h. To reconstruct the original Western blot lane, the beads were pooled, with the color IDs representing the molecular weight fraction of the proteins. For antibody incubation 5 µl of the DigiWest® bead mixes were added to 50 µl assay buffer (Blocking Reagent for ELISA (Roche) supplemented with 0.2% milk powder, 0.05% Tween-20 and 0.02% sodium azide) in a 96-well plate. In the next step, the assay buffer was discarded, 30 µl of primary antibody solution was added per well to the DigiWest® bead mixes and incubated overnight at 15 °C on a shaker (for primary antibody list, see SI Materials). Bead mixes were washed 2 × with PBST before adding 30 µl secondary antibody (labeled with phycoerythrin – PE) solution. After 1 h of incubation at 23 °C, the bead mixes were washed 2 × in PBST. Read-outs were performed using the Luminex FlexMAP 3D instrument. Protein bands were displayed as peaks by plotting the molecular weight against the corresponding median signal intensity. To integrate peaks of an expected molecular weight, a macro-based algorithm created in excel was applied. The local background was subtracted and for each peak the integral of the area was calculated (averaged fluorescent intensities – AFI). The resulting signals were normalized to total protein amount loaded onto the beads, if applicable centered on median of all BC-PDMs/PTT or only BC-PDMs samples. Subsequently, weak protein signals were determined as “lower detection limit minus one”. Further data processing is described in the figures.

### Drug testing in BC-PDMs using CellTox Green™ Cytotoxicity assay

To assess cell killing effects of different anti-cancer therapies and targeted therapies for breast cancer in BC-PDMs, the real-time CellTox™ Green Cytotoxicity assay (Promega) was performed according to manufacturer’s protocol. After the isolation of BC-PDMs from breast carcinoma specimen, the BC-PDMs were cultured for 1–2 weeks prior efficacy compound testing. The assays were performed according to manufacturer’s protocol. For each treatment, three to five replicates each with *n* = 15 BC-PDMs were prepared in phenol-red free BC-PDMs culture medium with a total volume of 150 µl. A proprietary cyanine dye binds to DNA in compromised cells leading to enhanced fluorescent signal. The dye is excluded from viable cells and thereby shows no increase in fluorescence. The fluorescent signal produced by the dye binding to DNA is therefore proportional to cell death. The dye was diluted 1:1000 and signals were measured as relative fluorescent unit (RFU) (485–500 nm Excitation / 520–530 nm Emission) using the Envision Multilabel Plate Reader 2102 and Tecan Spark Multimode Plate Reader. RFU values were background-corrected and treatment to DMSO (H_2_O) control fold changes were calculated for each measured time point. Outliers were excluded using Iglewicz and Hoaglin’s robust test for multiple outliers applying a recommended Z-score of ≥ 3.5 [27].

### Statistical analysis

Statistical analysis was performed using GraphPad Prism software. Statistical methods are illustrated in the respective figure legends. For Boxplot data, whiskers represent quartiles with minimum and maximum values and the median. Datasets with no normal distribution were analyzed with unpaired, two-tailed Mann–Whitney-U-test, otherwise as indicated. For all analyses, *p* values < 0.05 were considered statistically significant. Recommended post-hoc tests were applied for multiple comparisons. Data is analyzed as mean with standard error of the mean (SEM).

## Results

### BC-PDMs can be isolated from breast tumor tissues of different types with high viability

**Fig. 1:**
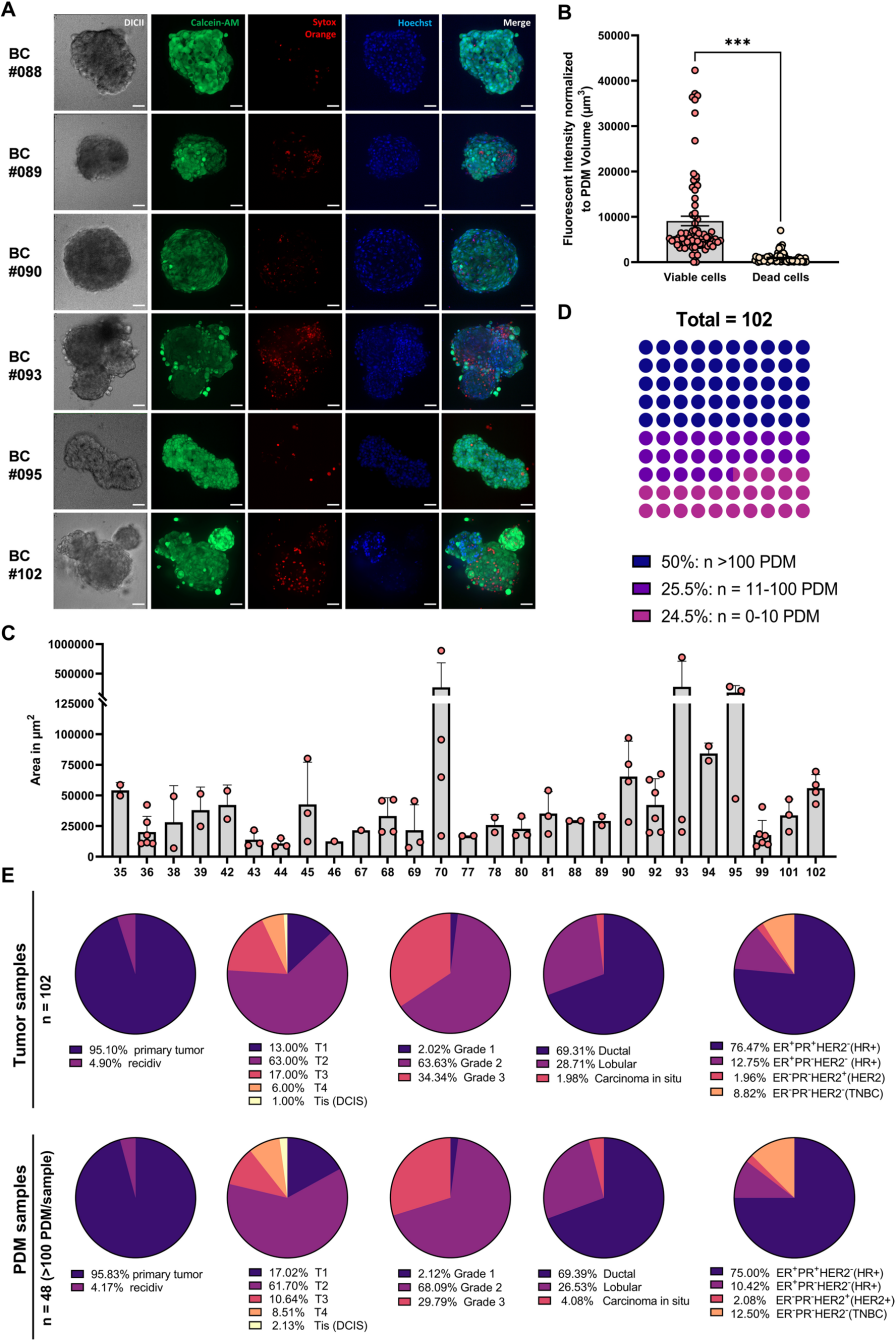
Isolation success of BC-PDMs. (**A**) Live-dead cell staining of isolated BC-PDMs from representative breast carcinoma tissue samples. BC-PDMs were stained with Calcein-AM (viable cells), SYTOX™ Orange (dead cells) and Hoechst 33258 (nuclei). Scale bars 50 µm. (**B**) Quantification of viable and dead cells in *n* = 27 BC models (on average three BC-PDMs per model) reveals high viability of BC-PDMs. Fluorescent intensities and volumes (µm3) were assessed using the Imaris Software. Wilcoxon paired signed rank test, ***p < 0.001. (**C**) Area measurement of BC-PDMs from *n* = 27 BC models. Data are shown as mean values with SD. (**D**) Success rate of microtumor isolation from *n* = 102 breast carcinomas. 50% of BC-PDMs reached a total number of more than 100 single microtumors. (**E**) Correlation of BC-PDM isolation success rate and clinical characteristics of corresponding breast carcinomas tissue samples

We previously established a novel 3D platform consisting of patient-derived microtumors (PDM) and tumor infiltrating lymphocytes (TILs) to identify treatment responses and therapeutic vulnerabilities in ovarian cancer and glioblastoma [23, 28, 29]. Here, we aimed to extend the PDM and TIL isolation (Figure S1) method to BC. Isolation and expansion of TIL populations was successful in > 95% of analyzed tissue samples with an average TIL viability of > 90% (Figure S1A-B). Multicolor flow cytometry analyses identified the presence of heterogenous subpopulations of regulatory and exhausted T cell populations (Figure S1C-H). The study enrolled patients over 18 years of age diagnosed with BC of all molecular subtypes. In total, we obtained *n* = 102 residual fresh mammary carcinoma tissue samples from debulking surgeries conducted at the University Hospital Tuebingen (Table S1). To analyze the viability of BC-PDMs after the isolation from BC specimen, we combined live-dead cell staining with 3D spinning disc confocal microscopy. As shown in Fig. 1A, viable cells were stained with Calcein-AM, dead cells with SYTOX™ Orange and nuclei with Hoechst dye. Comparing the fluorescent intensities of viable and dead cells normalized to the total measured volume (µm^3^) in *n* = 27 BC-PDMs models (Fig. 1B), the number of viable cells was significantly higher than that of dead cells (Wilcoxon signed rank test, *p* < 0.001). Within the *n* = 27 BC-PDMs samples, microtumors had variable sizes, with an average area of 59261 µm^2^, a maximum area of 888481 µm^2^ and a minimum area of 7003 µm^2^ (Fig. 1C, Table S2). The overall success rate of BC-PDMs isolation from *n* = 102 breast carcinomas was > 75%. We were able to isolate more than 100 PDM per sample from 50% of the tissue samples obtained (Fig. 1D). In 25.5% of cases, PDM were generated with less than *n* = 100 PDM per sample, while in the remaining 24.5%, no PDM were recovered from the tissue sample. In total, we successfully established *n* = 77 BC microtumor samples. Depending on the number of PDM recovered per sample, different downstream analyses could be performed such as immunohistochemistry (IHC), anti-cancer drug efficacy testing and/or protein profiling. To determine whether the success rate of BC-PDM isolation was related to specific clinical features of the original primary tumor, we correlated the available clinical data of the corresponding tumor samples and the obtained BC-PDM models (including samples with > 100 isolated PDM) (Fig. 1E). The success rate of BC-PDM isolation appeared to be largely independent of clinical features of the corresponding primary tumor tissue (PTT). BC-PDMs were successfully isolated from breast tumor tissue samples regardless of tumor grade, histological tumor type and hormone receptor status.

### Histotype-specific pathological characteristics of breast tumor tissue are conserved in corresponding BC-PDMs

**Fig. 2:**
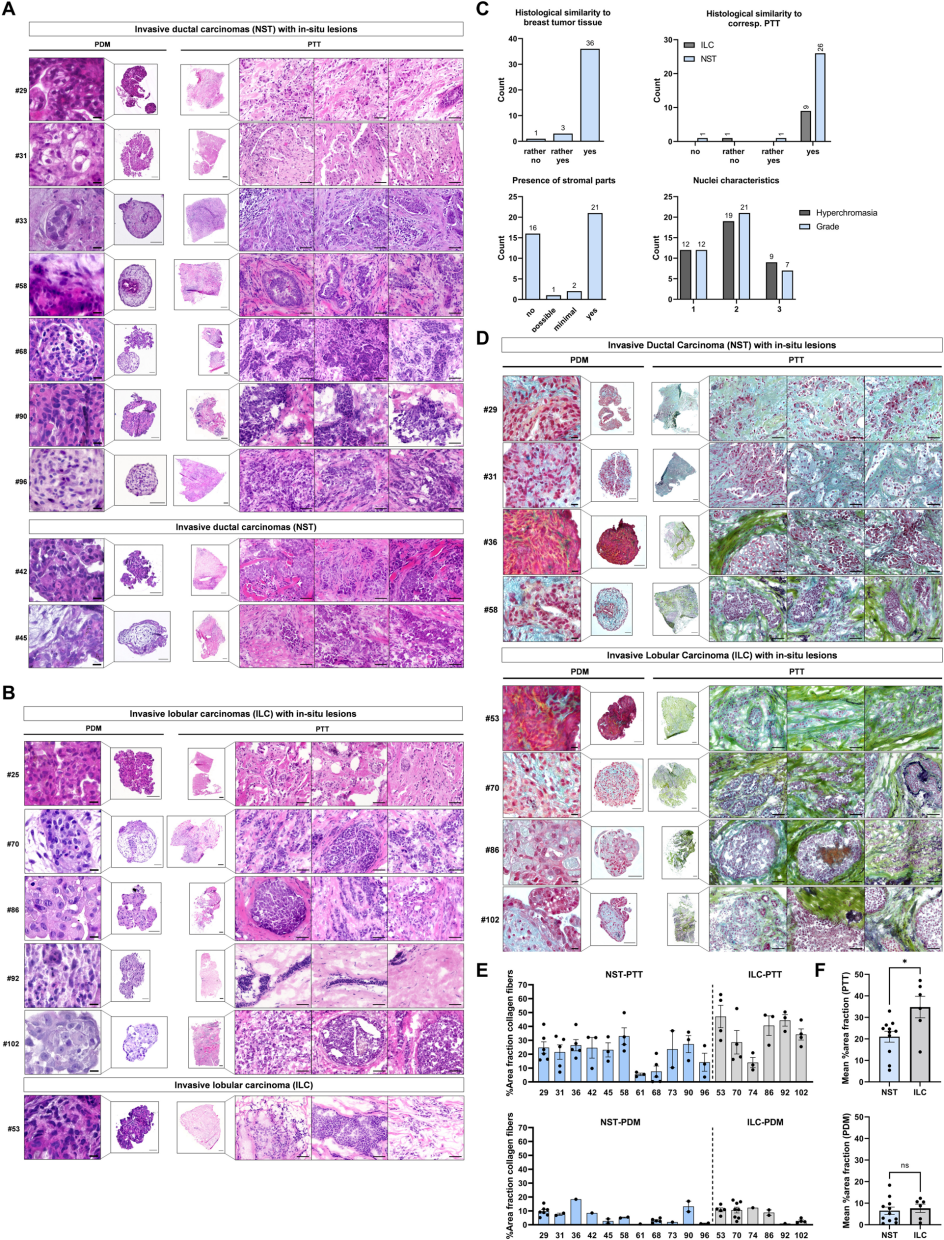
Histopathology and cytology of BC-PDMs and corresponding PTT. H&E staining of BC-PDMs and corresponding primary, (**A**) invasive ductal breast carcinomas (NST) with/without ductal in-situ (DCIS) lesions and (**B**) invasive lobular breast carcinomas (ILC) with/without lobular in-situ (LCIS) lesions. (**C**) Pathological evaluation of BC-PDMs. *n* = 39/40 BC-PDMs resembled histopathology of breast carcinomas, *n* = 36/38 of the corresponding primary tumor histotype (NST/ILC; NST/ILC histology not available for one sample; one other sample classified as medullary carcinoma and excluded from comparison of NST and ILC BC-PDMs) and *n* = 23/40 BC-PDMs displayed stromal parts. Histopathological tumor characteristics of BC-PDMs were assessed such as hyperchromasia and nuclei differentiation (nuclear grade 1: nuclei with little variation in size and shape; grade 3: large nuclei with high variation in size and shape; grade 2: nuclei show features between 1 and 3. (**D**) Movat-pentachrome staining revealed connective tissue compartments in BC-PDMs and PTT e.g. collagen fibers (yellow), PGs/GAGs (cyan blue), collagen/PGs/GAGs-superimposition (green), mucins (blue) and elastin (black; representative images shown for *n* = 8 matched pairs of BC-PDMs and corresponding PTT). (**E**) Amount of collagen fibers within BC-PTT and BC-PDMs. Collagen fibers are measured semi-quantitatively as %-area fraction. RGB images were unmixed by subtractive mixing (color deconvolution) via ImageJ. (**F**) Averaged %-area fraction of BC-PTT and BC-PDM (*n* = 17) samples shown in (**E**). Data are mean with SEM. *p < 0.05, **p < 0.01, ***p < 0.001. Unpaired, parametric t-test. Scale bars BC-PDMs: 50 µm/10 µm (zoom); PTT: 500 µm/50 µm (zoom; paired sections of BC-PDMs and corresponding PTT specimen were available from *n* = 17 samples displaying stromal parts for Movat-pentachrome stainings)

Breast carcinomas form a heterogenous group of tumors and show high variability in morphologic features, e.g. degree of pleomorphism, cellular atypia, mitotic activity or stromal circumference. Yet, there are morphological features characteristic of different histologic sub-types. Among others, tumor cells form nests, clusters, cords, trabeculae, or single file lines (“Indian File”) [30] depending on the specific sub-type. Using H&E staining, a certified pathologist compared the histopathological and cytological characteristics of the isolated BC-PDMs and the corresponding PTT. We divided the specimens according to histological classification into NST and ILC with or without in-situ components. Tumor cells of NST PTT formed irregular invasive nests/clusters, cords, and sheets within the stroma, in some tissues with glandular features (Fig. 2A). PTT further displayed distinct ascitic structures filled with tumor cells (#33, #68), tubular structures (#58, #68) with small lumina, papillary structures or no distinct architecture. Similar to corresponding PTT, tumor cells of NST BC-PDMs formed solid (cohesive), papillary nests with closely spaced cells (BC-PDMs/PTT: #33, #42, #58, #68, #90) and a clear separation from the ECM compartment. In addition, glandular structures were also evident within NST BC-PDMs (#31 and #45). The histopathologic architecture of ILCs with in-situ sites is more specific than that of IDC. The lobular ascites of in-situ lesions retained their overall structure in PTT and were filled with small, round, monomorphic epithelial cells almost without lumen (e.g. #70, #86). Infiltrating cells within ILCs were dispersed with poor cohesion and grew in slender strands or single files (so called “Indian Files”) or concentrically around ducts or lobules (PTT e.g. #25, #70, #86). Tumor cells of ILC BC-PDMs were mostly discohesive and dissociated in the surrounding stromal tissue (#25, #53, #86, #92, #102), thus resembling primary infiltrating tumor lesions (Fig. 2B). This histological feature was also found in NST BC-PDMs #96. Overall, pathological evaluation of BC-PDMs specimens revealed histological similarity to breast tumor tissue in 97.5% of cases (*n* = 39/40) and to histological tumor type (IDC/NST) in 95% of cases (*n* = 36/38) (Fig. 2C). Stromal compartments were present in 57.5% of cases (*n* = 23/40). In result of comparison of the cytopathology of BC-PDMs and corresponding PTT, similar cellular atypia was found. While some BC-PDMs consisted of small, rather homogenous cells without prominent nucleoli (e.g. #25, #29, #45, #53), other samples exhibited moderate (#31, #33, #58, #96) to strong nuclear pleomorphism (#68, #70, #86, #90, #92, #102) with large, hyperchromatic nuclei and prominent nucleoli. Most BC-PDMs resembled a moderate nuclear grade (*n* = 21) with moderate hyperchromasia (*n* = 19). While 20.5% (*n* = 8/39) of samples had a similar nuclear grade of BC-PDMs and corresponding PTT, the majority (59%) of BC-PDMs had a nuclear grade decreased by 1 degree (Figure S2A). In summary, BC-PDMs largely resemble the histopathology of the corresponding primary tumor tissue.

### BC-PDMs contain extracellular matrix components of the original tumor tissue

The ECM, representing a complex network of tissue fibers, glycoproteins (e.g. elastin, laminin, fibronectin), proteoglycans (PGs), and glycosaminoglycans (GAGs), not only provides stability and a reservoir for e.g. growth factors, but also plays a role in breast tumorigenesis, invasiveness [31, 32] and therapy response [33]. Furthermore, ECM stiffness and density were found to correlate with prognosis in breast cancer [34, 35]. To evaluate and compare the ECM within BC-PDMs and corresponding PTT, we used the Movat-pentachrome staining to visualize different components of connective tissue on a single slide [36]. In PTT sections, the predominant ECM components were PGs/GAGs (cyan blue) and collagen fibers (yellow), which mostly overlapped (green) (Fig. 2D). In all PTT, dense collagen networks were detected in close proximity to the tumor masses due to increased collagen deposition. This leads to the “stiffening” of the tissue [31]. The collagen fibers exhibited different morphologies: short and wavy (e.g. PTT #29), thin and linear (e.g. PTT #31) or thick and linear (e.g. #36, #53). Most notable were dense and thick collagen fibers wrapped around tumor masses (e.g. PTT #31, 58), especially in stromal areas adjacent to in-situ lesions (e.g. PTT #36, #86, #102). Tumor borders were either relatively smooth, with collagen fibers drawn at a tangential angle around the tumor (e.g. PTT #86) or oriented perpendicular in the direction of cell invasion (e.g. PTT #58) (Provenzano, 2006 #522). Corresponding BC-PDMs exhibited ECM components to a lesser extent compared to primary tissue. Despite limited enzymatic tissue disruption during BC-PDMs isolation with collagenase I and II, we detected collagen expression (yellow/green) in the corresponding BC-PDMs (e.g. BC-PDMs #29, #36, #58, #53, #70). Compared to tumor masses in the PTT, which are surrounded by thick collagen fibers, the arrangement of collagen in BC-PDMs was less specific. In BC-PDMs, the collagen rather formed a backbone structure for the tumor cells. In general, BC-PDMs appeared like small tumor fragments excised from tumor masses of the corresponding primary tumor tissue and consisted of the inner tumor cell mass with its ECM components, but without the framing collagen fibers. In addition to cross-linked collagen-fibers, PGs/GAGs (cyan blue) were found within tumor masses/islets of the PTT (e.g. #29, #58, #53, #70) and demarcated tumor masses from the stroma as a single layer separated from collagen fibers (e.g. PTT #58, #86, #102). PGs/GAGs were found in BC-PDMs when their expression within tumor masses in corresponding PTT was high (e.g., BC-PDMs #29, 31, #58). Elastic fibers (black) were mostly attached to collagen fibers (e.g. PTT #53, #86, #102) and were more abundant in ILC compared to IDC (NST) tissues. In contrast to other BC-PDMs, the ECM of BC-PDMs #102 exhibited elastic fibers, as in the corresponding primary tumor. Further, mucin (blue/gray) secreted by tumor cells was found in sections of PTT #31 and #86 and in the corresponding BC-PDMs. Different amounts of collagen were observed between ILC and NST tumors, both in PTT and PDM samples (Fig. 2E). Within ILC PTT, significant higher amounts of collagen fibers were detected compared to NST PTT (Fig. 2F), as previously reported [37]. Collagen deposition in PDM was reduced as compared to corresponding PTT sections as expected due to the restricted amount of BC-PDM available for these analyses. Data showed a non-significant trend towards higher collagen deposition in ILC BC-PDM (Fig. 2E-F). In conclusion, the Movat-pentachrome staining allowed the visualization of different ECM components of the primary tumor within BC-PDMs. Compared to whole tumor masses in tumor tissues, the ECM compartments in BC-PDMs occur to a lesser extent and in slightly different arrangement.

### Immunohistochemical analysis of hormone receptor expression enables distinction of BC-PDMs isolated from hormone receptor positive and TNBC primary tumors

**Fig. 3:**
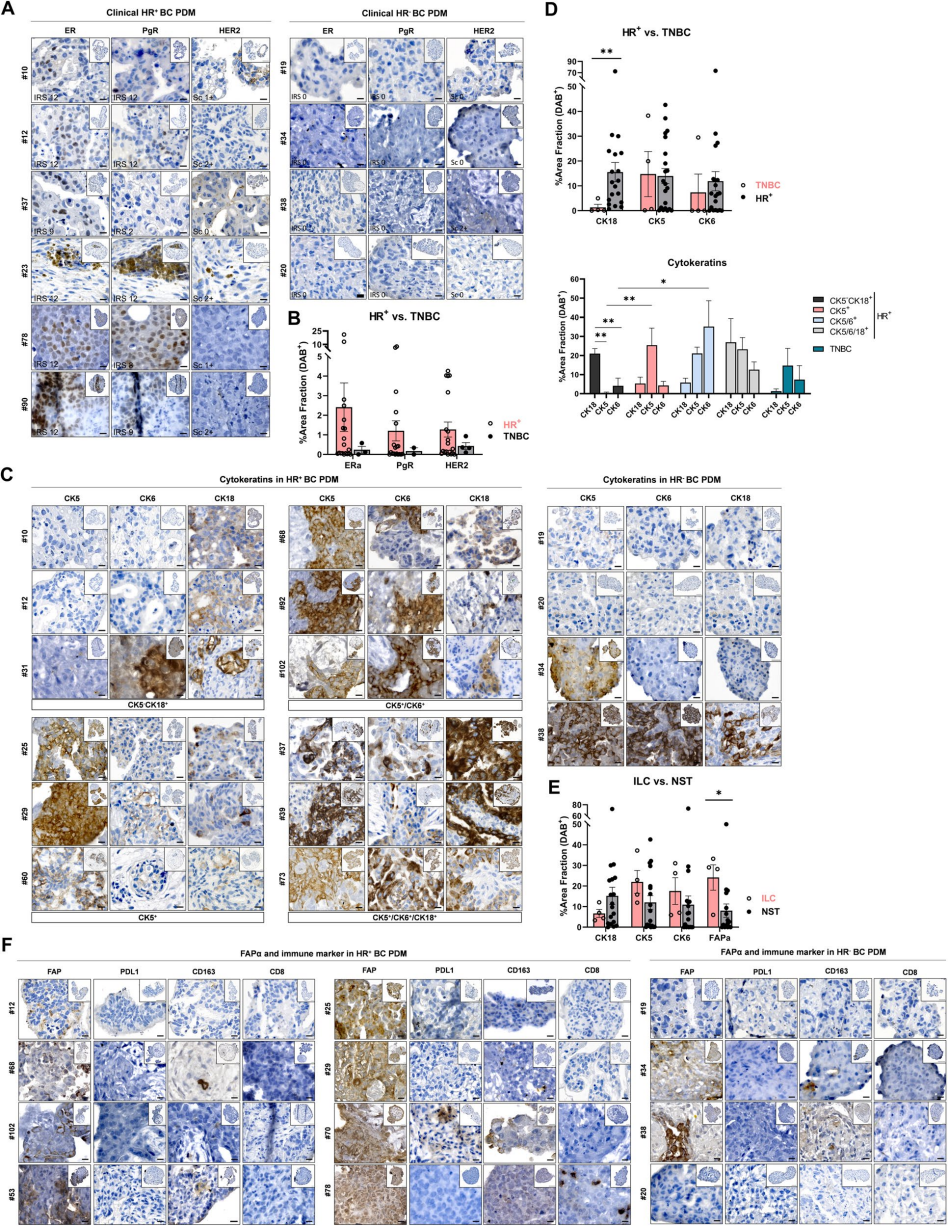
Immunohistochemical analysis of breast cancer specific and immune cell markers in BC-PDMs. DAB staining was analyzed semi-quantitatively as %-area fraction of a BC-PDMs. RGB images were unmixed by subtractive mixing (color deconvolution) using ImageJ software. (**A**) Hormone receptor (HR) DAB staining of clinically classified HR+ BC-PDMs vs. TNBC BC-PDMs. Clinically assessed immunoreactive scores (IRS) from primary tumor are indicated. HR+ BC-PDMs were arranged in ascending order of ERα expression (**B**) HR+ BC-PDMs have increased HR expression (ERα, PgR, HER2) compared to TNBC BC-PDMs. (**C**) DAB staining of luminal cytokeratin (CK18) and basal cytokeratins (CK5 and CK6). BC-PDMs were grouped into four groups according to CK staining: CK5^−^CK18^+^, CK5^+^, CK5/6^+^ and CK5/6/18^+^. (**D**) Significantly elevated expression of luminal CK18 vs. basal CK5/CK6 in HR+ compared to TNBC BC-PDMs. Mann–Whitney U test, **p = 0.006. Differences in CK18, CK5 and CK6 expression in HR+ and TNBC BC-PDMs according to their classification into the previously determined groups. Within group: One-way ANOVA, Holm-Šídák’s multiple comparisons test. Different group comparison: Two-way ANOVA, Holm-Šídák’s multiple comparisons test. (**E**) Differences in CK and FAPα expression in ILC BC-PDMs vs. NST BC-PDMs. NST BC-PDMs show higher levels of CK18, while ILC BC-PDMs show significant higher levels of FAPα. Mann–Whitney U-test, *p = 0.028. Both ILC/NST BC-PDMs express basal CK5 and 6. (**F**) DAB staining of FAPα and immune markers in BC-PDMs grouped into HR+ and TNBC. For HR+ BC-PDMs, BC-PDMs were arranged in ascending order of FAPα expression. Data are mean with SEM. *p < 0.05, **p < 0.01, ***p < 0.001. ERα: estrogen receptor alpha; PgR: progesterone receptor; HER2: HER2/neu-ErbB2 receptor

To further characterize BC-PDMs, we performed immunohistochemistry (IHC) analysis of FFPE BC-PDMs sections. We examined the expression of hormone receptors, cytokeratins as well as cancer-associated fibroblasts (CAFs) and immune cell markers using DAB staining. To analyze the expression of clinical molecular markers, we stained BC-PDMs sections for ERα, PgR and HER2. BC-PDMs were classified as hormone receptor positive (HR+) or triple negative (TNBC) as determined by pathologic evaluation of the primary tumor (Fig. 3A). TNBC is an aggressive type of BC usually with higher grade, higher rate of early recurrence and a worse 5-year prognosis [38–41]. It is defined by lacking expression of hormone receptors and HER2. For each tissue sample, the corresponding immunoreactive scores (IRS) and HER2 scores (0–3) were determined (Table S1). ERα and PgR staining of BC-PDMs was consistent with the corresponding clinical classification and was increased in BC-PDMs originating from HR+ PTT (Fig. 3B). The level of ERα and PgR expression varied within HR+ BC-PDMs. In contrast, HR expression was strongly reduced in TNBC PDMs. HER2 was detectable in HR+ BC-PDMs sample #10 and #37. However, HER2 expression in BC-PDMs #37 did not resemble its clinical HER2 score, which was reported to be zero. In conclusion, IHC staining enabled the identification of BC-PDMs isolated from clinical HR+ breast tumors and those isolated from clinical TNBC tumors based on hormone receptor expression.

### BC-PDMs display differential expression of luminal and basal cytokeratins

Since cytokeratin (CK) expression is thought to be stable throughout carcinogenesis [42], CKs are studied as differentiation markers in precancerous breast lesions. Breast tissue normally consists of a stratified epithelium with luminal epithelial cells surrounded by a basement membrane composed of myoepithelial cells, both with different CK phenotypes [43, 44]. Breast carcinomas are found to express different CKs, such as the luminal subtype expressing luminal epithelial CKs (CK8/CK18/CK19) or the basal subtype expressing basal myoepithelial high molecular weight (HMW) CKs (CK5/CK6/ CK7/CK14) [4, 45, 46]. Nevertheless, some breast tumors were shown to express both types of CKs [44, 47]. Here, we analyzed CK5, CK6 and CK18 staining of HR+ and TNBC PDMs. We found highly heterogenous staining of CKs in HR+ and TNBC PDM. The heterogenous CK expression allowed us to subdivide the BC-PDMs based on CK expression. Thus, we divided HR+ BC-PDMs into four groups based on the evaluated CK expression: CK5^−^/CK18^+^ (luminal, differential glandular phenotype), CK5^+^ (basal), CK5/6^+^ (basal, stem cell phenotype) and CK5/6/18^+^ (intermediate glandular phenotype) [48] (Fig. 3C). CK5^−^/CK18^+^-PDM showed significantly higher CK18 expression compared to CK5 (**p = 0.004) or CK6 (**p = 0.005) (Fig. 3D). The abundances of CK5 and CK6 were significantly higher in the CK5^+^ (*p* = 0.006) and CK5/6^+^ (*p* = 0.020) groups compared to the CK5^−^/CK18^+^ group. Some HR+ BC-PDMs were positive for all three CKs. Comparing the CK expression between HR+ BC-PDMs and TNBC PDM, we found significantly increased CK18 expression (*p* = 0.006), a marker for luminal carcinomas, in HR + BC-PDMs (Fig. 3D). TNBC BC-PDMs did not show CK18 expression, but moderate expression of CK5/6. This is consistent with the literature [44]. As a hallmark of EMT, lack of CK18 expression has been associated with tumor progression [49] as it promotes cancer cell migration [50]. Two of the four TNBC PDM analyzed here showed strong CK5 expression, and BC-PDMs #38 also displayed high CK6 expression. Due to high CK5/6 positivity correlating with poorer prognosis [51], TNBC PDM #38 was defined as a basal-like subtype of TNBC. Overall, CK5/6 expression was not significantly different among HR+ and TNBC PDMs (Fig. 3D). When ILC and NST BC-PDM were compared, ILC BC-PDM showed a non-significant trend towards higher expression of the HMW cytokeratins (CK5/6), whereas NST BC-PDM showed a non-significant trend towards higher expression of luminal CK18 (Fig. 3E). We next analyzed additional markers such as FAPα, associated with CAFs (cancer-associated fibroblasts), and immune cell markers CD163, CD8 and PD-L1 (Fig. 3F). PD-L1, a T cell inhibitory checkpoint marker, and CD8, a marker for cytotoxic T cells, were mostly absent from BC-PDMs except for BC-PDMs #70. CD8^+^ T cells were detected in BC-PDMs #78. Sporadic expression of CD163 indicating the presence of M2 macrophages was found in BC-PDMs (e.g. #68, #53, #70, #34). In contrast, FAPα was detectable in all stained BC-PDMs, to varying degrees. Among them, ILC BC-PDMs showed significantly stronger FAPα staining (Fig. 3E, *p* = 0.028) in accordance with the literature [52]. Significant differences between TNBC and HR+ BC-PDMs were not identified. In conclusion, BC-PDMs largely reflect the hormone receptor status of the corresponding tumor tissue and exhibit heterogeneous expression of CKs and FAPα, which are markedly different in HR+ and TNBC and ILC/NST BC-PDMs. In addition, immune cell markers could be identified sporadically in BC-PDMs and independent of hormone receptor status.

### Protein expression and signaling pathway activity of BC-PDMs correlate with corresponding primary tumors

**Fig. 4:**
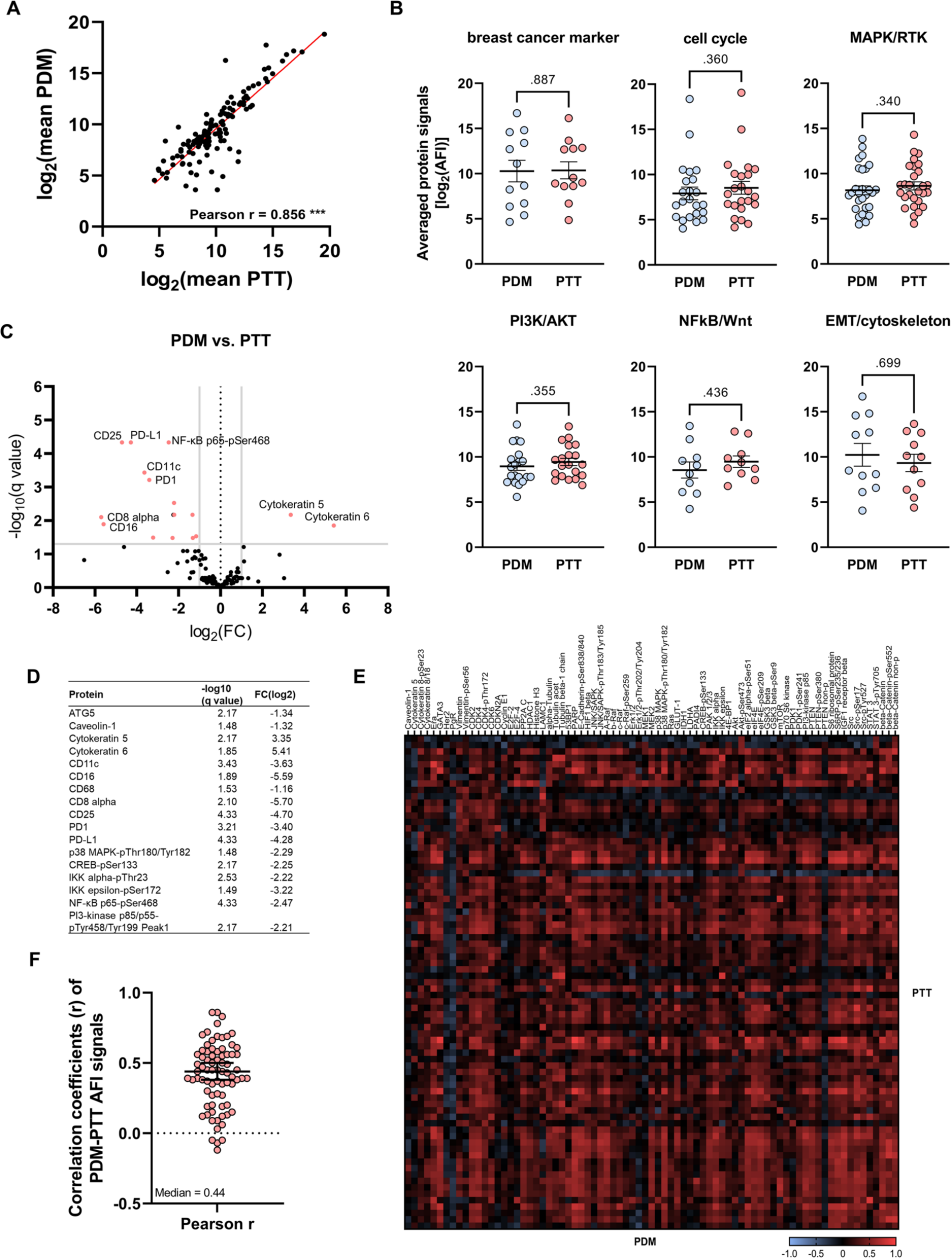
Comparison of protein profiles from BC-PDMs and corresponding primary tumor tissue. *N* = 20 matched BC-PDM and PTT-pairs were analyzed. (**A**) X–Y plot of correlated protein means of BC-PDMs and PTT. Protein signals of measured BC-PDM-PTT samples were correlated using Pearson correlation. DigiWest AFI protein signals were averaged for BC-PDMs/ PTT and log_2_ transformed. Each dot represents one protein. Pearson *r* = 0.856; ***p < 0.001. (**B**) Overall signaling pathway activity in BC-PDMs resembled that of primary BC tumors. Proteins were sorted by pathway affiliation. Shown are AFI protein signals, averaged for BC-PDMs/PTT and log_2_ transformed. Mann–Whitney test; *p* values as indicated. (**C**-**D**) Differently expressed proteins of matched BC-PDMs-PTT samples. Volcano plot shows proteins with significantly decreased or increased expression in BC-PDMs (red) with an adjusted FDR *p*-value (-log10 (q)) > 1.3 and a log_2_ fold change >|1|; multiple t-test with Welch correction; Benjamini, Krieger, and Yekutieli FDR. Exact values are shown in (**D**). (**E**) Heatmap of unclustered pearson correlation coefficients (r) shows moderate correlation of AFI protein signals over BC-PDMs and matched PTT samples. (**F**) Pearson correlation coefficients (r) displayed as scatter plot with a median correlation of *r* = 0.44. Data are mean with SEM. AFI: averaged fluorescent intensities

Following histological characterization, we extended the comparison of BC-PDMs with corresponding primary tumor tissues by in-depth quantitative protein profiling analyses. We therefore measured protein expression and activity of key signal transduction pathways in *n* = 20 matched BC-PDM-PTT pairs employing the DigiWest® technology [26]. In this way, we generated protein profiling datasets covering 142 total and phosphorylated proteins (raw data: Table S3; BC-PDM-PTT data: Table S4). The analyzed profiling panel comprised proteins from the cell cycle, Jak/STAT, MAPK, RTK, PI3K/Akt, EMT/cytoskeleton and Wnt signaling pathways. Pearson correlation revealed an overall high, positive correlation of averaged protein signals between matched BC-PDMs and PTT with P_r_ = 0.856 (*p* < 0.001; Fig. 4A). Furthermore, comparison of signaling pathway activity and expression of breast cancer-related proteins, resulted in no significant differences. Overall, the average protein expression of BC-PDMs resembled that of matched breast cancer tissue (Fig. 4B, Table S5). Subsequently, changes in protein abundance were determined between BC-PDMs and PTT pairs. In total, *n* = 18 analytes displayed significant differences in expression (-log10 (q) > 1.3) and a log_2_ fold change of at least |1| (Fig. 4C-D). BC-PDMs had increased protein levels of the cytoskeletal protein cytokeratin 5 and 6 (CK5/6), while expression of immune cell markers CD11c, CD16, CD68, CD8 alpha, CD25, PD1 and PD-L1 were decreased. This is consistent with our IHC data, demonstrating that BC-PDMs are small tumor fragments composed of tumor cells, ECM proteins and partially stromal cells of the corresponding tumor tissue, with immune cell infiltrates in a few cases. Other proteins displaying reduced expression in BC-PDMs as compared to matched PTT belong to different signaling pathways. Among them were mainly phospho-proteins of the MAPK pathway (p38 MAPK-pThr180/Tyr163), the PI3K pathway (PI3K p85/p55-pTyr458/199) and the NFkB pathway (NFkB p65-pSer172, IKK alpha-pThr23, IKK epsilon-pSer172). Correlation data of individual proteins showed a general, positive correlation between protein signals of matched BC-PDMs/PTT-pairs (Fig. 4E) with a median coefficient of *r* = 0.44 (Fig. 4F). Table S6 lists the proteins whose signal levels correlated significantly with those of the primary tumors. Significant positive correlations were found across all signaling pathways. Among them, ERα protein expression was significantly correlated between BC-PDMs and matched PTT (*r* = 0.86, ***p < 0.001). ERα is clinically relevant for the classification of breast tumors. In addition to the histological assessment, we demonstrated at the protein level that the protein signaling pathway profiles of BC-PDMs are similar to those of the original tumor tissue across several signaling pathways. In individual cases, differences between the results of protein profiling analyses and histopathological assessment were observed. The corresponding tumor of BC-PDM #81 was classified as TNBC according to histopathology, whereas the result of protein profiling identified this model as ERα positive. In contrast, tumor sample #36 showed expression of ER according to histopathology, but not according to protein profiling. Overall, protein expression of PTT is reflected in BC-PDMs with high correlation.

### Cross-comparison of protein profiling data among individual BC-PDMs identifies personalized pathway activation signatures

**Fig. 5:**
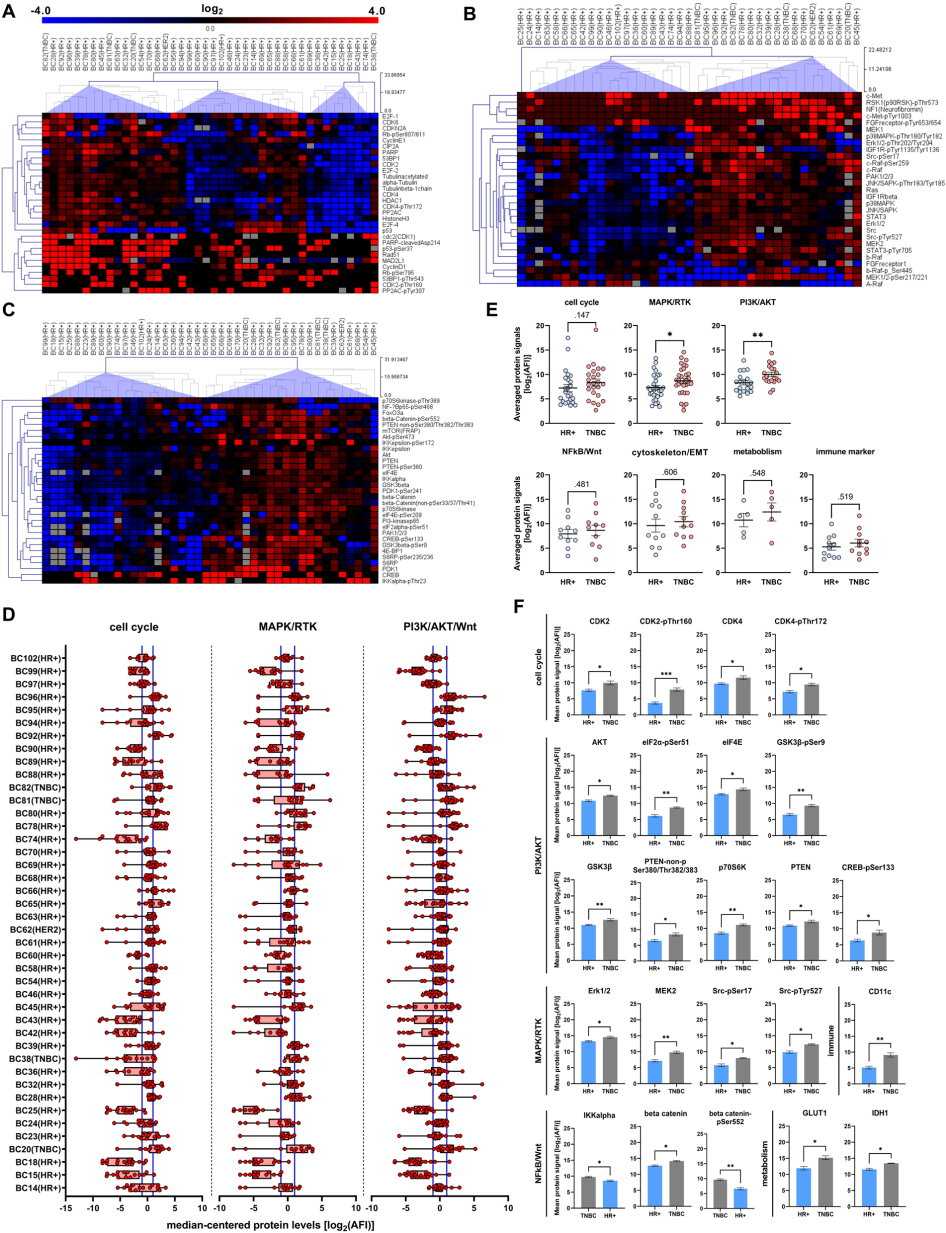
DigiWest-based protein pathway profiling of BC-PDMs. Hierarchical cluster linkage analysis (HCL) of median-centered, log_2_ transformed AFI protein signals of *n* = 42 BC-PDMs, divided into cell cycle, MAPK/RTK and PI3K/Akt pathways. Molecular subtype classifications of BC-PDMs as indicated. (**A**) HCL of sample and cell cycle-related analytes with complete linkage. Four sample clusters were identified based on differential expression levels. (**B**) HCL of sample and MAPK/RTK-related analytes with average linkage. There are two main sample clusters (excl. BC-PDM #25) that separate samples with high MAPK/PI3K protein expression from those with low expression. (**C**) HCL of sample and PI3K/AKT-related analytes with complete linkage. Two main sample clusters were identified: “high-expression” and “low-expression”. (**D**) Differences in signal transduction in BC-PDMs samples. Box-whisker plots show median-centered, log_2_ transformed AFI protein signals of different pathways. Data distribution within samples is illustrated by lines connecting min. and max. values. Each red dot represents a protein. Black lines in box plots indicate the “median” of measured proteins within a sample. Blue lines delineate the values >|1| corresponding to a fold change > 0.5. (**E**) TNBC BC-PDMs showing elevated PI3K/AKT- and MAPK/RTK- pathway activity. The averaged, log_2_ transformed protein signals are compared between TNBC and HR+ BC-PDMs within different pathways. Mann–Whitney U test, PI3K: *p* = 0.006, MAPK/RTK: *p* = 0.032. (**F**) Differentially expressed proteins in TNBC BC-PDMs. Comparison of mean protein expression in TNBC vs. HR+ BC-PDMs. Enhanced protein abundances in TNBC BC-PDMs were found for several proteins associated with cell cycle, metabolism, immune system, PI3K/AKT, MAPK/RTK and NFkB pathway. Mann–Whitney U test, *p < 0.05, **p < 0.01, ***p < 0.001. Data are mean with SEM

To classify the BC-PDMs samples based on their individual protein profiles, we analyzed signaling pathway activity of *n* = 42 BC-PDMs samples using hierarchical cluster linkage (HCL) analysis (Fig. 5; Table S7). In addition, samples were assigned according to clinical data as HR+ , TNBC or HER2-positive (HER2+) (illustrated in Table S1). Cluster analysis of cell cycle-related proteins resulted in four sample groups with different levels of cell cycle regulator expression (Fig. 5A). In addition to cluster 1, which included the BC-PDM sample #38, all HR+ BC-PDM samples with either weak or mixed expression levels were grouped into cluster 2 (*n* = 8) and 3 (*n* = 17). Clusters differed mainly in the expression of transcriptional activators E2F-1, E2F-2, transcriptional repressor E2F-4 and p53. TNBC, HER2+ and the remaining HR+ BC-PDM samples were grouped into cluster 4 (*n* = 16) and showed overall increased expression of cell cycle regulatory proteins. HCL of MAPK-RTK pathway proteins distinguished three sample groups separating *n* = 19 HR+ BC-PDMs with overall decreased protein abundances from *n* = 19 TNBC, HER2 + and HR+ BC-PDMs with elevated expression levels (Fig. 5B). Notably c-Met, RSK1-pThr573, NF1 and c-Raf were upregulated in the latter group compared with the HR+ -only group. When comparing PI3K/Akt pathway activity among individual BC-PDM models, samples were divided into two groups, too, with one group again consisting of HR + samples and the other containing all TNBC and HER2+ samples (Fig. 5C). Here, BC-PDMs were characterized by enhanced levels of beta-catenin, FoxO3a, Akt-pSer473, CREB, CREB-pSer133, PDK1 and IKKalpha-pThr23.

Next, we visualized the median-centered protein profiling data of BC-PDMs in box-whisker plots. This allowed us to identify individual BC-PDMs samples with increased expression of proteins belonging to cell cycle, MAPK/RTK and/or PI3K/AKT signaling pathways, respectively (Fig. 5D). Of interest were BC-PDMs samples with median-centered protein expression log_2_ AFI ≥ 1, corresponding to a fold change ≥ 2 (Table S8). Upregulated cell cycle activity was identified in *n* = 8 BC-PDMs, whereas MAPK/RTK signaling was amplified in *n* = 11 BC-PDMs with median expression levels ≥ 1. Higher PI3K/Akt pathway activity was present in *n* = 7 BC-PDMs. Interestingly, all three signaling pathways were concomitantly upregulated in the four BC-PDMs samples #20, #78, #92 and #96. At the same time other BC-PDM models showed simultaneous downregulation of all analyzed signaling pathways as indicated by log_2_ AFI values ≤ -1 (e.g. BC-PDMs #15, #18, #60, #89, #99). Pathway analysis thus allowed the classification of individual BC-PDM samples based on specific protein expression profiles. Histopathologic phenotypes were not observed to correlate with pathway activity.

### TNBC-PDMs exhibit increased PI3K/AKT and MAPK/RTK pathway activity

DigiWest-based protein profiling of BC-PDMs also enabled the differentiation of TNBC PDM from HR+ BC-PDMs. TNBCs are known to be characterized by altered oncogenic signaling pathways such as PI3K/Akt and MAPK/Erk [53]. Genetic aberrations of upstream regulators, such as activating mutations of PI3K, Ras, b-Raf, loss of function mutations of PTEN, overexpression of EGFR, have been shown to be common in breast cancer and play an important role in its dysregulation [54–59]. These changes can cause the development of chemoresistance in TNBC patients [60–62]. In line with these findings, we found PI3K/Akt (*p* = 0.006) and MAPK/RTK (*p* = 0.032) pathways significantly upregulated within TNBC PDM as compared to HR+ BC-PDMs (Fig. 5E). Proteins with significantly elevated abundance included AKT (*p* = 0.022), eIF2α-pSer51 (*p* = 0.009), eIF4E (*p* = 0.049), GSK3beta (*p* = 0.006), GSK3beta-pSer9 (*p* = 0.007), PTEN (*p* = 0.040), PTEN non-p (*p* = 0.044), p70S6K (*p* = 0.009), CREB-pSer133 (*p* = 0.041). All these regulators have previously been associated with TNBC. Furthermore, we were able to assign additional proteins with elevated abundance in TNBC PDM to the MAPK/RTK pathway. Parallel to the PI3K signaling, the MAPK pathway is another driving force in TNBC [63] and correlates with high disease recurrence rates in patients with TNBC [64]. We observed significant upregulation for Erk1/2 (*p* = 0.022), MEK2 (*p* = 0.002), Src-pSer17 (*p* = 0.012) and Src-pTyr527 (*p* = 0.014) (Fig. 5F). Other signaling pathways (e.g. cell cycle, NFkB-Wnt) did not show a significant distinction in expression between TNBC and HR+ BC-PDMs. However, we identified upregulation of individual proteins related to the cell cycle: CDK2 (*p* = 0.022),

CDK2-pThr160 (*p* < 0.001), CDK4 (*p* = 0.025) and CDK4-pThr172 (*p* = 0.019). While CDK2 hyperactivation is linked to basal-like breast cancer tumors [65], aberrant expression of CDK4 is linked to drug resistance [66]. Consistent with increased eIF2α-phosphorylation in TNBC PDM and the associated upregulation of aerobic glycolysis [67–69], we also found an upregulation of metabolism-related proteins including GLUT1 (*p* = 0.029) and IDH1 (*p* = 0.029). When comparing BC-PDMs derived from NST and ILC tumors, we detected no differences in overall signaling pathway activity (Figure S3A). However, we observed differential expression for individual proteins such as E-Cadherin-pSer838/840, CK8-pSer23 and ERα (Figure S3B). Decreased E-Cadherin levels in ILC BC-PDMs are in accordance with inactivating *CDH1* (E-Cadherin) mutations that are frequently observed in ILC tumors and disrupt cellular adhesion/epithelial integrity [70, 71]. In accordance with Ciriello et al. [72], we discovered lower GATA 3 protein levels in ILC tumors. Reduced ERα signal in ILC BC-PDMs may be explained by decreased GATA3 expression, as it plays a pivotal role in the recruitment of the ER transcription complex [73]. In summary, identified overexpressed signaling proteins in TNBC PDM affect many different cellular processes in cancer cells, including proliferation, differentiation, migration, cell growth and survival. Our results are consistent with previous findings in TNBC and show that BC-PDMs reflect protein signaling pathway activation characteristics of corresponding primary breast tumors.

### Identification of marker panels for individualized responses towards hormone- and chemotherapy using combined cytotoxicity and protein profiling analyses of BC-PDMs

**Fig. 6:**
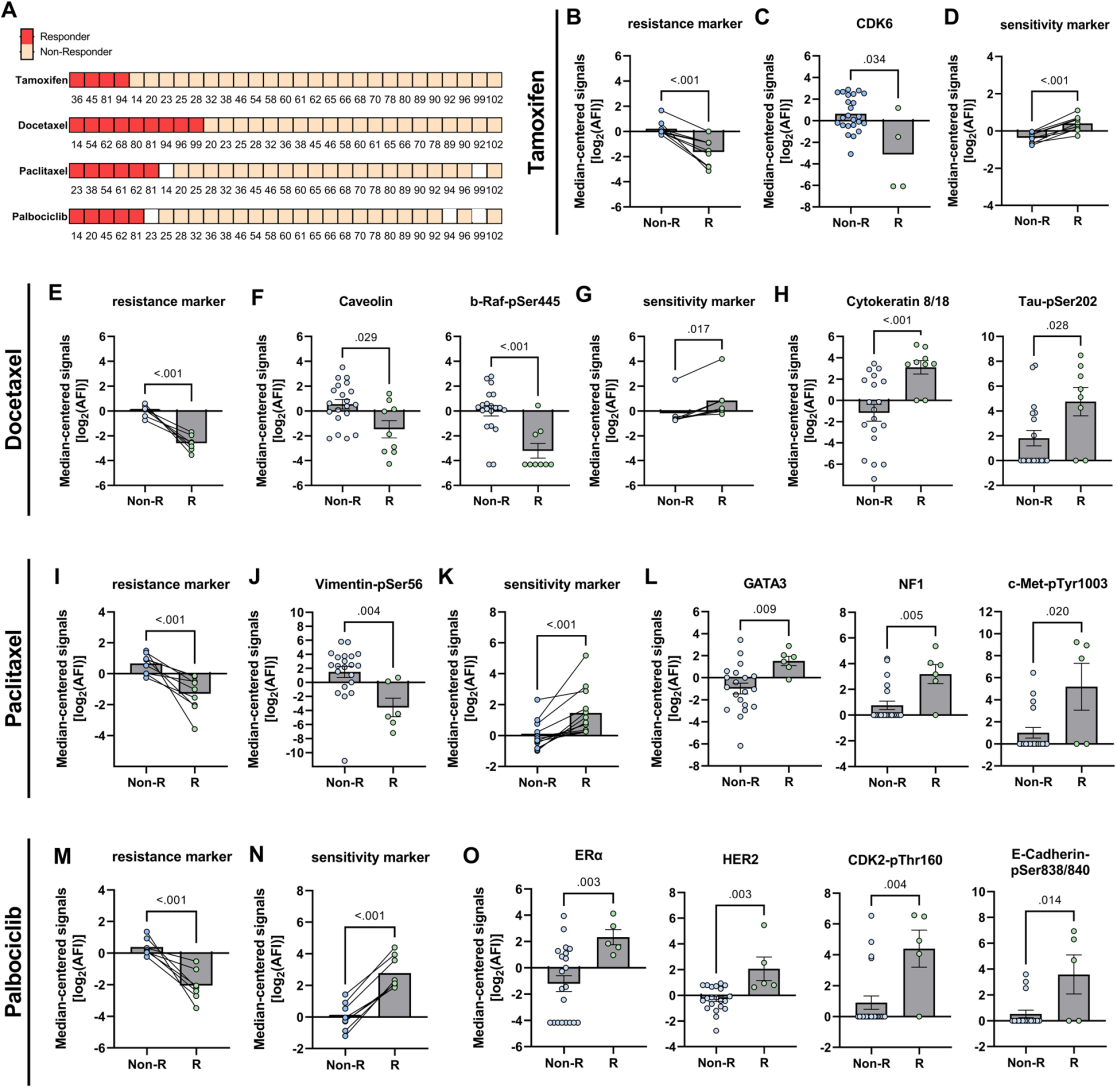
Treatment responses analyzed in BC-PDMs and identification of resistance and sensitivity marker panels. (**A**) Treatment response of breast cancer (BC) PDM to anti-cancer drugs. Microtumors were classified as “responder” and non-responder” based on the results of cytotoxicity measurements (Celltox Green™ assay; Promega). Cytotoxicity was determined in a time series (24 h, 48 h and 72 h). Treatment effects were analyzed as fold change of the respective control for each measurement time point using a mixed-effects model (REML) and Fisher’s uncorrected LST test. Statistically significant fold changes were defined as “response” and BC-PDMs were accordingly classified as “responders”. The numbers indicate BC sample number. (**B**-**D**) TAM, (**E**–**H**) DTX, (**I**-**L**) PTX and (**M**–**O**) PAB resistance and sensitivity marker panels. Median-centered, log_2_-transformed DigiWest AFI protein signals were compared between R and Non-R groups. Each data point within the scatter bar plots represents the same protein in R and Non-R. Lines connect protein data points between Non-R and R. Therapy resistance and sensitivity panels were identified including up to thirteen proteins (for detailed protein list see Table 1). Comparison of R and Non-R protein “panel” signals by non-parametric, unpaired Mann–Whitney U test. Within these protein panels individual, differentially expressed proteins are depicted (non-parametric, unpaired Mann–Whitney U test). *p < 0.05, **p < 0.01 and ***p < 0.001. Shown are mean with SEM. AFI: average fluorescent intensities; Non-R: non-responder; R: responder; TAM: tamoxifen (100 nM), DTX: docetaxel (5.5 µM), PTX: paclitaxel (4 µM), PAB: Palbociclib (150 nM)

BC-PDMs responses to four anti-cancer drugs were evaluated by a microplate-based cytotoxicity assay. Microtumors derived from different patients were treated with the selective estrogen receptor modulator [74] tamoxifen (TAM), the taxane chemotherapeutics docetaxel (DTX) and paclitaxel (PTX), and the CDK4/6 inhibitor palbociclib [75]. Samples were not differentiated according to receptor status since differences regarding the receptor status determined by histopathology and by protein profiling analysis, respectively, were observed in individual cases (see Protein expression and signaling pathway activity of BC-PDMs correlate with corresponding primary tumors section). Treatment-induced cell death was measured in a time series (24 h, 48 h and 72 h) and compared to the respective vehicle control (Table S9). A significant treatment effect, defined as a significant fold change in cell death between vehicle control and treatment, was considered a response, whereas a nonsignificant effect was considered a non-response or treatment resistance. (Mixed-effects model, Fisher’s uncorrected LSD test). This approach allowed to divide the samples into responder (R) and non-responder (Non-R) groups (Fig. 6A). BC-PDMs responded heterogeneously to the applied drug treatment. Most frequently they responded to treatment with DTX (9/29). Four samples showed a response to TAM (4/29), six samples to PTX (6/29) and five samples to PAB (5/29). Next, we compared the protein expression profiles (median-centered, log_2_ transformed data) of the previously determined responder and non-responder BC-PDM groups. Using DigiWest® analysis, we generated resistance/sensitivity protein marker panels that clearly distinguished responder from non-responder BC-PDMs (Fig. 6). For each treatment, we selected proteins that are associated with therapy response/resistance according to literature and are significantly differentially expressed in responder vs. non-responder BC-PDMs or are involved in therapy-related signaling pathways (Table 2, Figure S4).

**Table 2:**
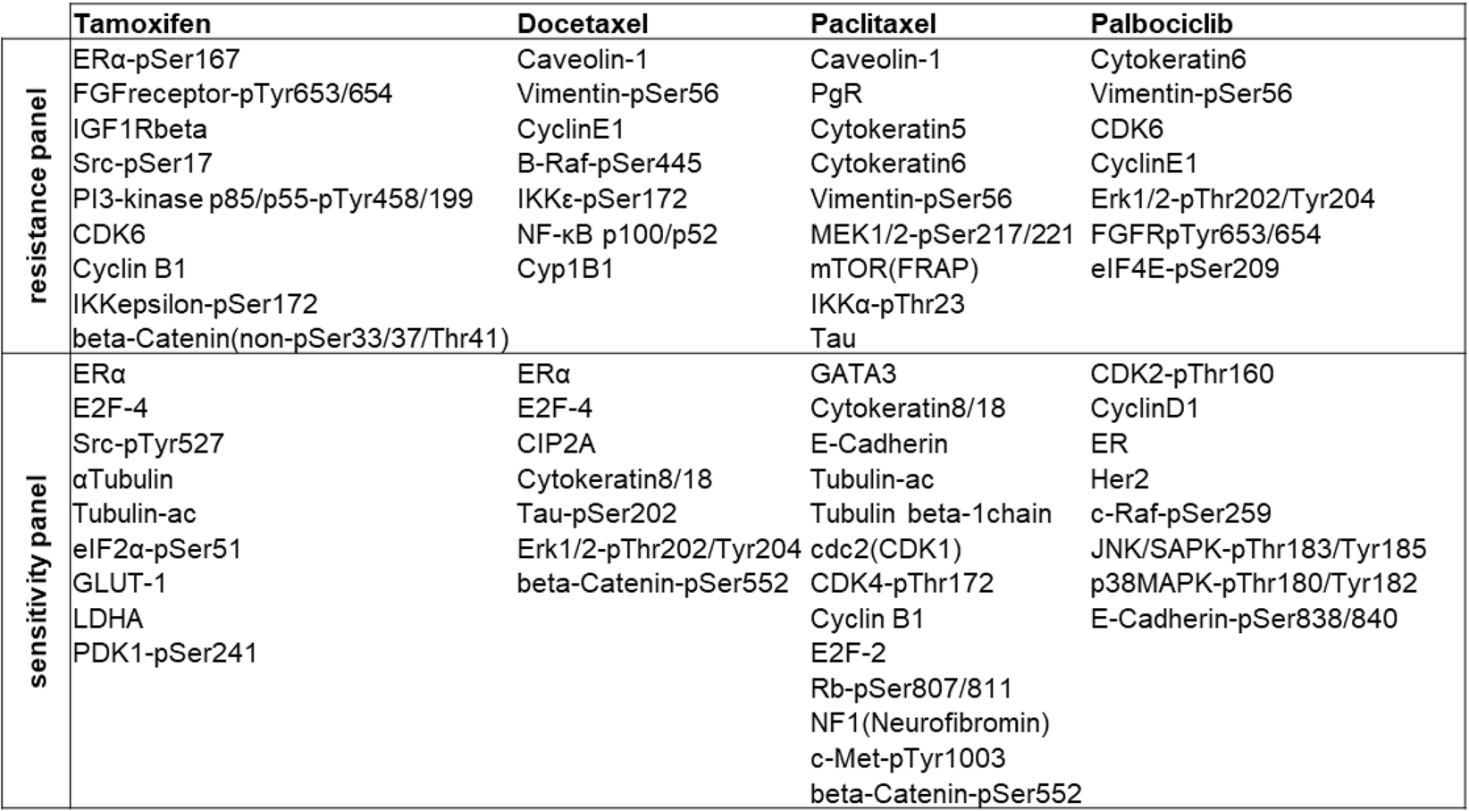
Treatment-resistance and -sensitivity panel of BC derived microtumors

In the TAM responder group, we identified a panel of nine proteins with significantly decreased abundances (Fig. 6B, Mann Whitney U test, ***p < 0.001). Phosphorylated proteins that were elevated in the treatment-resistant BC-PDM group (Table 2) included ERα-pSer167, FGFR-pTyr653/654, PI3-kinase p85/p55-pTyr458/199, and IKKepsilon-pSer172, all of which are directly or indirectly related to TAM resistance according to the literature [76–81]. The panel further contained regulators of the cell cycle (CDK6, Cyclin B1) and the Wnt-signaling pathway (non-phosphorylated beta-catenin). Within this panel, CDK6 expression was significantly different in non-responder versus responder BC-PDMs (Fig. 6C, Mann–Whitney U test, *p = 0.035). In simple logistic regression analysis, CDK6 was found to negatively affect the likelihood of response to TAM with a 50% decrease in the odds (OR = 0.5, 95% CI 0.21–0.82) (Figure S4B; Table S10; *p* < 0.05 [Wald, LRT]). A panel of nine proteins with increased abundance was found to correlate with TAM sensitivity (Fig. 6D, Mann Whitney U test, ***p < 0.001). This included ERα, the transcriptional repressor protein E2F-4, the microtubule protein αTubulin and proteins involved in cancer cell metabolism (GLUT1, LDHA and PDK1-pSer241) and stress responses (eIF2A-pSer51).

Using a 7-protein resistance panel, we were able to significantly distinguish DTX non-responder from DTX responder BC-PDMs (Fig. 6E, Mann Whitney U-test, ***p < 0.001). This panel included proteins associated with EMT induction (Vimentin-pSer56, NFκB p100/p52 and IKKε-pSer172) or drug metabolism (CYP1B1), which are also known to induce drug resistance to DTX and PTX in cancer cells [82, 83] (Table 2). In addition, higher Caveolin-1, Cyclin E1 and b-Raf**-**pSer445 protein levels contributed to DTX resistance of BC-PDMs. We found Caveolin-1 (*p = 0.029) and the MAPK-pathway related protein b-Raf**-**pSer445 (***p < 0.001) to be significantly enriched in non-responder BC-PDMs (Fig. 6F, Mann Whitney U-test). Figure 6G shows the protein panel predicting sensitivity of BC-PDMs to DTX treatment (Mann Whitney U-test, *p = 0.017) with increased expression of e.g. ERα, luminal-cell marker (CK8/18), inactive beta-catenin-pSer552 and microtubule associated protein Tau-pSer202 (Table 2). In this panel, we identified CK8/18 (Fig. 6H, Mann Whitney U-test, ***p < 0.001) and Tau-pSer202 (Fig. 6H, Mann Whitney U-test, *p* = 0.028) to be significantly enriched. By logistic regression analysis, expression of Caveolin-1 and b-Raf**-**pSer445 was shown to decrease the odds of DTX response of BC-PDMs by 44% (OR = 0.56, 95% CI: 0. 0.32–0.88) and 54% (OR = 0.46, 95% CI: 0.25 to 0.72) and thus contribute to DTX resistance. In contrast, elevated Tau-pSer202 (OR = 1.46, 95% CI: 1.06 to 2.22) and CK8/18 (OR = 1.54, 95% CI: 1.06 to 2.67) levels were significantly associated with DTX treatment response in BC-PDM (Figure S4D; Table S10; *p* < 0.05 [Wald, LRT]).

Paclitaxel treatment resistance of BC-PDMs was determined by a heterogenous panel of 9 proteins enriched in non-responder BC-PDMs (Fig. 6I, Mann Whitney U-test, ***p < 0.001). Resistance-associated proteins were Caveolin-1, PgR, mTOR, phosphorylated MEK1/2 (pSer217/221) of the Erk/MAPK signaling pathway, phosphorylated IKKα (pThr23) of the NFκB pathway, the microtubule-associated protein Tau and the basal breast cancer markers CK5, CK6 and Vimentin-pSer56 (Table 2). Moreover, we identified Vimentin-pSer56 to be significantly enriched in the PTX non-responder BC-PDMs (Fig. 6J, Mann Whitney U-test, **p = 0.004). Using a 13-protein panel, we could differentiate PTX sensitive from resistant BC-PDMs (Mann Whitney U-test, ***p < 0.001). We discovered several cell cycle-associated proteins (e.g. CDK1, CDK4-pThr172), luminal epithelial cell markers (e.g. E-Cadherin, CK8/18), the microtubule-forming protein Tubulin (acetylated Tubulin, Tubulin beta-chain), the Ras-inhibitor NF1 (Neurofibromin), c-Met-pTyr1003 and beta-Catenin-pSer55, whose expression affected BC-PDMs sensitivity to PTX treatment. Protein abundances differed significantly for GATA3 (Fig. 6L, Mann Whitney U-test, **p = 0.009), NF1 (Fig. 6L, Mann Whitney U-test, **p = 0.005) and c-Met-pTyr1003 (Fig. 6L, Mann Whitney U-test, *p = 0.020). The probability of BC-PDM response to PTX was doubled by increased GATA3 (OR = 2.34, 95% CI: 1.24–6.2) and NF1 (OR = 2.15, 95% CI: 1.25–4.5) expression and decreased levels of Vimentin-pSer56 (OR = 0.72, 95% CI: 0.51–0.93) (Figure S4F; Table S10; *p* < 0.01 [Wald, LRT]). To the best of our knowledge, there are no studies to date that have reported a link between the expression of these proteins and PTX treatment response.

For PAB treatment, we identified a resistance panel including proteins previously associated with PAB resistance: CDK6, Cyclin E1 and FGFR. Combined with basal breast cancer markers CK 6 and Vimentin, the MAPK-signaling protein Erk1/2- pThr202/Tyr204 and the active mTOR signaling protein eIF4E-pSer209, these proteins could differentiate PAB resistant from PAB sensitive BC-PDMs (Fig. 6M, Mann Whitney U-test, ***p < 0.001). In contrast, sensitivity to PAB was predicted by a 8-protein panel (Fig. 6N and O, Mann Whitney U-test, ***p < 0.001) with increased ERα (**p = 0.003), HER2 (**p = 0.003), CDK2-pThr160 (**p = 0.004), E-Cadherin-pSer838/840 (*p = 0.014), Cyclin D1, c-Raf-p259, JNK/SAPK-pThr183/Tyr185 and p38MAPK-pThr180/Tyr182 signals in responder BC-PDMs. An increase of ERα (OR = 2.15, 95% CI: 1.2–5.91), HER2 (OR = 72.48, 95% CI: 2.36–14,948,598) and E-Cadherin-pSer838/840 (OR = 1.84, 95% CI: 1.15 to 3.55) by one level more than doubled the odds of BC-PDMs responding to PAB therapy (Figure S4H; Table S10; *p* < 0.01 [Wald, LRT]).

In summary, we identified heterogeneous responses to anti-cancer drug treatment in BC-PDMs. Through comprehensive molecular protein signaling pathway analysis of treatment-responsive and -resistant BC-PDMs, we gained insights into the treatment response mechanisms of breast cancer cells in microtumors, which were shown to resemble histopathological and protein expression profile characteristics of the corresponding primary breast tumor. Our data confirmed several proteins known to play a role in treatment resistance and/or sensitivity, and also identified novel markers that significantly correlate with individualized treatment responses.

## Discussion

Breast cancer is a highly heterogenous disease with profound morphological, genetic and phenotypical variability resulting in multiple disease manifestations with different response to treatment [16]. Gene expression analysis and classical immunohistochemical analysis has enabled the differentiation of BC subtypes and subsequently served to guide treatment selection and patient stratification in BC [4–6]. Still, development of treatment resistance remains a major challenge in the management of this malignancy, largely due to the pronounced intra-tumoral heterogeneity that characterizes BC beyond genetic profiles [16]. Apart from the intrinsic changes and interactions of tumor cells, also the crosstalk of tumor cells with the complex TME impacts the BC phenotypic manifestation and thus the development of treatment resistance [19, 20]. In this context, the use of tumor models accurately representing the complexity of patient tumors, while at the same time being applicable for a variety of readout methods, is becoming increasingly important. To date, a number of different ex vivo platforms and model systems have been described in this context, such as patient-derived tumor organoids, tumor explants, tumor slices, and others [84–86]. In this study, we successfully generated a repertoire of microtumor samples from different BC subtypes representing disease heterogeneity. We applied previously published protocols for isolating microtumors from primary tumor tissues [23, 28, 29]. BC-PDMs recapitulate general histological features and tumor-type specific features of NST (IDC) and ILC like growth patterns, cellular pleomorphism and atypia of the corresponding primary tumor tissue. Using Movat-pentachrome stainings, we found the most abundant BC-related ECM proteins [37, 87], collagen and PGs/GAGs, also present in BC-PDMs and show a tendency for increased collagen deposition within ILC-type PDM comparable to PTT. Studies demonstrated that collagen deposition, which increases ECM stiffness, and the density and orientation of collagen fibers affect tumor aggressiveness, invasiveness, therapy responses and correlates with prognosis in BC [88–90]. Hence, BC tumor models that comprise ECM structures of native tumors like BC-PDMs represent relevant test systems to investigate disease biology and therapy resistance.

Moreover, our results highlight other features in BC-PDMs characteristic of different BC subtypes as previously described, including hormone-receptor expression in HR+ BC-PDMs compared with TNBC -PDMs, increased collagen deposition in ILC derived BC-PDMs [37], heterogenous expression profiles of luminal (CK18) and basal cell markers (CK5 and CK6) [44] with decreased CK18 expression in TNBC -PDMs [49], and high FAPα expression in ILC BC-PDMs [52]. Regarding the CK expression in BC-PDMs, we observed similar cellular profiles as described previously by Abd El-Rehim, D.M. et al. [44], i.e. the differentiated glandular phenotype (CK18^+^), the stem cell phenotype (CK5/6^+^) and an intermediate glandular phenotype (CK5/6^+^, CK18^+^) [48]. In contrast to this study, we did further differentiate CK5^+^ from CK5^+^/CK6^+^ BC-PDMs. According to several reports, 17% of ILCs express basal CKs [91]. In our study, ILC BC-PDMs expressed relatively high levels of CK5/6 compared to NST BC-PDMs, which is therefore somewhat surprising. In order to provide a more precise statement on this, BC-PDMs established from a larger cohort of ILC samples would need to be evaluated. However, differential protein expression analysis revealed an overall higher expression of CK5 and CK6 in BC-PDMs regardless of breast tumor type compared to primary tumors. Overexpressed CK5 could be attributable to low estrogen concentrations during culture of BC-PDMs as described before [92–94]. Similar results have been observed in organoids derived from BC patients [95]. We cannot exclude the possibility that the culture conditions for BC-PDMs may favor the selection and outgrowth of BC subclones with a basal epithelial phenotype (CK5/CK6^+^), which are often associated with *BRCA-1* mutated BC [96, 97] and are underrepresented in the primary tumor. This observation warrants further investigation in future studies.

Compared to frequently employed gene expression analysis of tumor models, our study investigated BC microtumors on the protein level using the DigiWest® method covering 142 total and phosphoproteins. Thereby, breast cancer-related protein expression levels and signaling pathway profiles largely correlated with those of corresponding primary tumors. Hierarchical cluster analysis grouped BC-PDMs according to their classification and molecular protein expression signature. Further, our DigiWest® data confirmed protein signatures of TNBC-PDMs consistent with those in the literature, characterized by upregulated PI3K/Akt and MAPK/RTK signaling [53, 63, 64, 98] with overexpressed proteins associated with integrated stress response [99–102], higher relapse rates, mortality [64, 103, 104], tumor growth and EMT [29, 105–107]. When comparing BC-PDMs and primary tumor profiles, we found decreased expression of NFkB signaling pathway proteins NFkB regulates processes of immune and inflammatory responses and is part of the immune defense against transformed cells [108, 109]. Because the protein data also showed diminished expression of immune cell markers in BC-PDMs, the attenuated presence of immune cells in microtumors might explain the observed, decreased NFkB-related signals as compared to PTT.

Our study validates the application of BC-PDM for in vitro functional drug testing, as demonstrated previously for ovarian cancer and glioblastoma microtumors [23, 28, 29], to functionally complement molecular and histopathological analyses. Protein profiling analysis combined with functional drug testing allowed us to identify phenotypic hallmarks of treatment resistance and sensitivity, as opposed to genetic alterations that may not correlate with clinical benefit [21]. As the growth of some types of BC is driven by increased signaling from estrogen and progesterone receptors, hormone therapies have been developed that prevent hormones from binding to these receptors. TAM is a competitive inhibitor of the estrogen receptor known as a selective modulator, while fulvestrant is a selective ER degrader [110]. It has been reported that overexpressed CDK6 inhibits fulvestrant-mediated (ER-down regulation-induced) apoptosis and thus induces fulvestrant-resistance [111]. Our data implicates that TAM resistance may also be characterized by high CDK6 levels in BC-PDMs illustrating the possibility of resistance mechanisms similar to fulvestrant. Furthermore, it is known that ERα activation through phosphorylation of Ser167 in an estrogen-independent manner and FGFR activation can cause TAM resistance: both proteins were identified within our BC-PDMs TAM resistance panel [76, 80, 112]. In line with the clinical application of TAM in HR+ BC [113], increased total ERα levels contribute to TAM sensitivity in BC-PDMs.

Drug treatment assays with BC-PDMs were conducted independently of hormone receptor status. In individual cases, differences in the result of histopathological analysis of hormone receptor expression in tumor tissue compared to protein analysis were evident. Residual fresh tumor tissue specimens received for BC-PDM isolation and protein expression analyses were inherently not identical to the sample of the corresponding tumor tissue examined by histopathology. Breast carcinoma is characterized by marked intratumoral heterogeneity, with consequences previously described in the literature, including reduced concordance rates in receptor expression between core and excisional biopsies [114–117]. In addition, a minority of approximately 10% ER-negative breast carcinomas together with a molecularly defined subset of TNBC have been described in the literature to show response to tamoxifen [118, 119].

The chemotherapeutic agent DTX has shown high activity as an antimicrotubular agent in both neoadjuvant and adjuvant application in advanced and metastatic breast cancer [120]. It also had the strongest effect on BC-PDM treatment response as compared to other anti-cancer drugs tested. In line with previous studies, BC-PDMs generated from less invasive BC, luminal-like CK8/18 high BC-PDMs with inactive β-catenin signaling and thus lower EMT-transition, and BC-PDMs with high ER expression were sensitive to treatment [121–123]. Contrary, we confirmed that high expression of EMT-related and EMT–inducible proteins, high expression of DTX-metabolizing CYP1B1 and increased Caveolin-1 in BC-PDMs predict DTX resistance [82, 83, 124, 125]. Surprisingly we did identify Ser202 phosphorylated Tau to positively and b-Raf-pSer445 to negatively influence DTX sensitivity of BC-PDMs. To date, there are no reports on either protein or their potential impact on response to taxane treatment. However, there are conflicting data on whether the expression of Tau correlates with taxane response [126, 127].

As the first taxane compound discovered, PTX has a similar function to DTX as antimicrotubular agent [128]. The critical role of the EMT process in PTX resistance, [83], is well represented indicated by the resistance and sensitivity marker panel we identified in BC-PDMs, including EMT-regulator proteins such as Vimentin-pSer56, CK5, CK6, E-Cadherin, CK8/18, IKKα-pThr23, beta-Catenin-pSer55. Contrary to DTX, our results regarding PTX resistance of BC-PDMs indicate that increased total au protein levels correlate with treatment resistance. Further studies are warranted to further investigate the importance of Tau protein expression in taxane treatment response of breast cancer. In line with previous in vitro studies our data suggest a correlation between high PgR levels and decreased PTX sensitivity [129]. Interestingly, we found three proteins being significantly elevated in PTX sensitive BC-PDMs: GATA3, NF1 and c-Met-pTyr1003. So far, these proteins have not been linked to taxane sensitivity, but have generally been associated with breast cancer development [130–133].

In addition to endocrine and chemotherapy, we also tested the CDK4/6 inhibitor palbociclib [75]. The emergence of several intrinsic and acquired resistance mechanisms has been described preclinically, however without verification in the clinical setting [134]. Our comparison of responder and non-responder BC-PDM protein expression profiles provided intriguing results regarding PAB treatment. We identified several proteins in our BC-PDM resistance/sensitivity panel to be predictive for PAB response that have been linked to PAB resistance/sensitivity in previous studies, such as CDK6, Cyclin E1, FGFR, Cyclin D1 and ERα [134]. Surprisingly, our data also suggest Vimentin, CK6, CDK2-p and HER2 protein expression as novel PAB-treatment response markers. Increased Vimentin and CK6 levels may define a more aggressive and invasive tumor type that is resistant to PAB [51, 135]. Our analyses identified phosphorylated CDK2 to contribute to PAB-sensitivity of BC-PDMs, while other studies reported the opposite, as the cyclin E-CDK2 pathway is an important bypass mechanism of the cyclin D1-CDK4/6 axis in acquired PAB-resistance [134]. Both CDK4/6-Cyclin D and CDK2-Cyclin E complexes are decisive for the transition of G1- to S-phase and thus required for cell cycle progression. Further studies are warranted to evaluate this differential response in BC-PDMs.

In summary, we have shown that a salient feature of BC-PDMs, in addition to their histopathological and molecular similarity to the corresponding patient tumor, is the representation of native ECM components that collectively represent the disease heterogeneity of BC. Limitations of this novel patient-derived model system are the restricted number of microtumors available for downstream analyses, the reduced expression of immune cell markers and NFkB signaling proteins, as well as the enhanced expression of CK5 and CK6 as compared to corresponding primary tumor tissue. Further evaluation in additional sample cohorts will be needed to understand the underlying mechanism and to assess the long-term stability of HR-expression in BC-PDMs cultures. In this context, a subtype-specific analysis of drug treatment effects in BC-PDMs, with a particular focus on TNBC cases, would also be of special interest for future studies. Moreover, the application of BC-PDMs in patient-derived xenograft mouse models would allow the study of long-term growth kinetics as well as processes of tumor metastasis as recently described [136]. Regarding the application of BC-PDMs for assessment of immune cell interaction and immune-oncological treatment responses, we have previously shown functional drug testing of immune checkpoint inhibitors in co-cultures of ovarian cancer and glioblastoma PDM and autologous immune cells [23, 28, 29].

## Conclusion

Based on comprehensive protein profiling analyses in combination with functional drug testing assays in BC-PDMs our study highlights the potential of identifying patient-tumor specific, differentially expressed proteins to discriminate treatment responders from non-responders and warrants further, confirmatory studies in larger sample cohorts. Specifically, future studies will focus on the comparison of functional drug testing and protein profiling data from BC-PDMs with clinical treatment response in respective patients. As a complement to genomic mutation analysis and standard subtype classification, the combination of individual histopathologic analysis, preclinical drug testing, and parallel protein profiling analyses of BC-PDMs may hold promise for identifying predictive markers of treatment resistance and sensitivity to personalize breast cancer therapies.

### Supplementary Information

**Additional file 1: SI Materials 1.** Antibodies used in DigiWest protein profiling analysis. **Figure S1.** Multicolor flow cytometry analysis of isolated and expanded TILs from BC specimen. **Figure S2.** Correlation of nuclear grade in PDM and PTT. **Figure S3.** Proteomic comparison of NST and ILC-derived BC PDM. **Figure S4.** Identification of resistance and sensitivity marker panels in treatment responder and non-responder microtumors and regression analysis of differently expressed proteins. **Table S1.** Clinical patient data of the patient cohort. **Table S2.** Descriptive statistics of “area” and “fluorescent intensity live/dead” measurements in PDM. **Table S3.** Raw data of DigiWest® protein signals in PDM and PTT samples and total measured protein amounts. **Table S4.** DigiWest®-based AFI protein signals in matched PDM-PTT pairs DigiWest®-based AFI protein signals in matched PDM-PTT pairs. **Table S5.** DigiWest®-based AFI protein signals of matched PDM-PTT pairs sorted by pathway affiliation. **Table S6.** Pearson correlation of protein abundances in PDM and corresponding PTT (PDM/PTT pairs). **Table S7.** DigIWest®-based AFI protein signals of *n* = 42 PDM samples. **Table S8.** Descriptive statistics of averaged, median-centered and log_2_ transformed protein signals for cell cycle, MAPK/RTK and PI3K/AKT pathway in *n* = 42 PDM samples. **Table S9.** Celltox™ Green assay RFU (relative fluorescent unit) values of BC microtumors treated with TAM, DTX, PTX and PAB. **Table S10.** Descriptive statistics of simple logistic regression analysis of differentially expressed proteins in treatment responder and non-responder groups.

## Data Availability

The data that support the findings of this study are available from the corresponding authors upon reasonable request and after signature of an MTA from the corresponding authors.

## Abbreviations

BC: Breast cancer
CAFs: Cancer-associated fibroblasts
CK: Cytokeratin
DAB: 3,3′-Diaminobenzidin
DCIS: Ductal carcinoma in-situ
DMSO: Dimethyl sulfoxide
DTX: Docetaxel
ECM: Extracellular matrix
EMT: Epithelial-to-mesenchymal transition
ER: Estrogen receptor
FAPα: Fibroblast-associated protein α
FC: Fold change
FFPE: Formalin-fixed paraffin-embedded
GAGs: Glycosaminoglycans
H&E: Hematoxylin and eosin staining
HCL: Hierarchical clustering
HER2: Human epidermal growth factor receptor 2
HR: Hormone receptor
IDC: Invasive ductal carcinoma
IHC: Immunohistochemistry
ILC: Invasive lobular carcinoma
IRS: Immunoreactive score
LCIS: Lobular carcinoma in-situ
NST: Invasive ductal carcinoma of no special type
PAB: Palbociclib
PDM: Patient-derived microtumors
PgR: Progesterone receptor
PGs: Proteoglycans
PTT: Primary tumor tissue
PTX: Paclitaxel
RFU: Relative fluorescent units
TAM: Tamoxifen
TIL: Tumor infiltrating lymphocytes
TME: Tumor microenvironment
TNBC: Triple-negative breast cancer
