# Supplementary material for: Breast cancer patient-derived microtumors resemble tumor heterogeneity and enable protein-based stratification and functional validation of individualized drug treatment
Source: J Exp Clin Cancer Res. 2023 Aug 18;42:210. doi: 10.1186/s13046-023-02782-2 (PMC10436441; doi:10.1186/s13046-023-02782-2)
Supplement: Supplementary file 1 — Additional file 1: SI Materials 1. Antibodies used in DigiWest protein profiling analysis. Figure S1. Multicolor flow cytometry analysis of isolated and expanded TILs from BC specimen. Figure S2. Correlation of nuclear grade in PDM and PTT. Figure S3. Proteomic comparison of NST and ILC-derived BC PDM. Figure S4. Identification of resistance and sensitivity marker panels in treatment responder and non-responder microtumors and regression analysis of differently expressed proteins. Table S1. Clinical patient data of the patient cohort. Table S2. Descriptive statistics of “area” and “fluorescent intensity live/dead” measurements in PDM. Table S3. Raw data of DigiWest® protein signals in PDM and PTT samples and total measured protein amounts. Table S4. DigiWest®-based AFI protein signals in matched PDM-PTT pairs DigiWest®-based AFI protein signals in matched PDM-PTT pairs. Table S5. DigiWest®-based AFI protein signals of matched PDM-PTT pairs sorted by pathway affiliation. Table S6. Pearson correlation of protein abundances in PDM and corresponding PTT (PDM/PTT pairs). Table S7. DigIWest®-based AFI protein signals of n = 42 PDM samples. Table S8. Descriptive statistics of averaged, median-centered and log2 transformed protein signals for cell cycle, MAPK/RTK and PI3K/AKT pathway in n = 42 PDM samples. Table S9. Celltox™ Green assay RFU (relative fluorescent unit) values of BC microtumors treated with TAM, DTX, PTX and PAB. Table S10. Descriptive statistics of simple logistic regression analysis of differentially expressed proteins in treatment responder and non-responder groups. [file 13046_2023_2782_MOESM1_ESM.zip › Anderle et al_Revised Supplementary Information_final_ESM.docx]

**Supplementary Information**

**SI Materials:**

SI Materials 1. Antibodies used in DigiWest protein profiling analysis.

**Supplementary Figures:**

Figure S1. Multicolor flow cytometry analysis of isolated and expanded TILs from BC specimen

Figure S2. Correlation of nuclear grade in PDM and PTT.

Figure S3. Proteomic comparison of NST and ILC-derived BC PDM.

Figure S4. Identification of resistance and sensitivity marker panels in treatment responder and non-responder microtumors and regression analysis of differently expressed proteins.

Supplementary Tables:

Table S1. Clinical patient data of the patient cohort.

Table S2. Descriptive statistics of “area” and “fluorescent intensity live/dead” measurements in PDM.

Table S3. Raw data of DigiWest® protein signals in PDM and PTT samples and total measured protein amounts.

Table S4. DigiWest®-based AFI protein signals in matched PDM-PTT pairs DigiWest®-based AFI protein signals in matched PDM-PTT pairs.

Table S5. DigiWest®-based AFI protein signals of matched PDM-PTT pairs sorted by pathway affiliation

Table S6. Pearson correlation of protein abundances in PDM and corresponding PTT (PDM/PTT pairs).

Table S7. DigIWest®-based AFI protein signals of n = 42 PDM samples.

Table S8. Descriptive statistics of averaged, median-centered and log2 transformed protein signals for cell cycle, MAPK/RTK and PI3K/AKT pathway in n = 42 PDM samples.

Table S9. Celltox™ Green assay RFU (relative fluorescent unit) values of BC microtumors treated with TAM, DTX, PTX and PAB.

Table S10. Descriptive statistics of simple logistic regression analysis of differentially expressed proteins in treatment responder and non-responder groups.

**SI Materials**

SI Materials 1. Antibodies used in DigiWest protein profiling analysis.

| **Analyte** | **Uniprot** | **Product_No** | **Supplier** |
| --- | --- | --- | --- |
| 4E-BP1 | Q13541 | 1557-1 | Abcam |
| 53BP1 | Q12888 | 4937 | Cell Signaling |
| 53BP1-pThr543 | Q12888 | 3428 | Cell Signaling |
| Akt | P31749 | 4685 | Cell Signaling |
| Akt-pSer473 | P31749 | 4060 | Cell Signaling |
| alpha-SMA | P62736 | 14968 | Cell Signaling |
| alpha-Tubulin | P68366 | 302211 | Synaptic Systems |
| A-Raf | P10398 | 4432 | Cell Signaling |
| ATG5 | Q9H1Y0 | 12994 | Cell Signaling |
| Beclin-1 | Q14457 | 3738 | Cell Signaling |
| beta-Catenin | P35222 | 8480 | Cell Signaling |
| beta-Catenin(non-pSer33/37/Thr41) | P35222 | 8814 | Cell Signaling |
| beta-Catenin-pSer552 | P35222 | 9566 | Cell Signaling |
| b-Raf | P15056 | 14814 | Cell Signaling |
| b-Raf-p-Ser445 | P15056 | 2696 | Cell Signaling |
| BRCA1 | P38398 | 9010 | Cell Signaling |
| Caveolin-1 | Q03135 | 3238 | Cell Signaling |
| CD11c | P20702 | 45581 | Cell Signaling |
| CD16 | P08637 | 80006 | Cell Signaling |
| CD25 | P01589 | 13517 | Cell Signaling |
| CD3epsilon | P07766 | 4443 | Cell Signaling |
| CD4 | P01730 | PA5-87425 | Thermo Fisher Scientific |
| CD56 | P13591 | 3576 | Cell Signaling |
| CD68 | P34810 | 86985 | Cell Signaling |
| CD8alpha | P01732 | 85336 | Cell Signaling |
| cdc2(CDK1) | P06493 | 9112 | Cell Signaling |
| CDK2 | P24941 | 2546 | Cell Signaling |
| CDK2-pThr160 | P24941 | 2561 | Cell Signaling |
| CDK4 | P11802 | 12790 | Cell Signaling |
| CDK4-pThr172 | P11802 | PA-64482 | Invitrogen |
| CDK6 | Q00534 | sc-7961 | Santa Cruz |
| CDKN2A | P42771 | 10883-1-AP | Protein Tech Group |
| CIP2A | Q8TCG1 | A301-454A | Bethyl |
| c-Met | P08581 | 3148 | Cell Signaling |
| c-Met-pTyr1003 | P08581 | 3135 | Cell Signaling |
| c-Raf | P04049 | 9422 | Cell Signaling |
| c-Raf-pSer259 | P04049 | 9421 | Cell Signaling |
| CREB | P16220 | 9197 | Cell Signaling |
| CREB-pSer133 | P16220 | 9198 | Cell Signaling |
| CTMP | Q96KR2 | 4612 | Cell Signaling |
| CyclinD1 | P24385 | 2978 | Cell Signaling |
| CyclinE1 | P24864 | 4129 | Cell Signaling |
| Cyp1B1 | Q16678 | sc-374228 | Santa Cruz |
| Cytokeratin5 | P13647 | M3270 | Spring Bioscience |
| Cytokeratin6 | P02538 | 2302-1 | Abcam |
| Cytokeratin8/18 | P05783 | 4546 | Cell Signaling |
| Cytokeratin8-pSer23 | P05787 | 2147-1 | abcam (Epitomics) |
| E2F-1 | Q01094 | 3742 | Cell Signaling |
| E2F-2 | Q14209 | DR1095-100UG | Millipore |
| E2F-4 | Q16254 | orb10571 | Biorbyt |
| E-Cadherin | P12830 | sc-59778 | Santa Cruz |
| E-Cadherin-pSer838/840 | P12830 | 2239-1 | Abcam |
| eIF2alpha-pSer51 | P05198 | 3398 | Cell Signaling |
| eIF4E | P06730 | 2067 | Cell Signaling |
| eIF4E-pSer209 | P06730 | 9741 | Cell Signaling |
| ER | P03372 | RM-9101-S | Thermo Fisher Scientific |
| ERalpha-pSer167 | #NV | 2514 | Cell Signaling |
| Erk1/2 | P28482 | 4695 | Cell Signaling |
| Erk1/2-pThr202/Tyr204 | P28482 | 4370 | Cell Signaling |
| FGFreceptor1 | P11362 | 9740 | Cell Signaling |
| FGFreceptor-pTyr653/654 | P11362 | 3476 | Cell Signaling |
| FLOWER(C9orf7) | Q9UGQ2 | orb164624 | Biorbyt |
| FoxO3a | O43524 | 2497 | Cell Signaling |
| GATA3 | P23771 | 5852 | Cell Signaling |
| GLUT-1 | P11166 | 07-1401 | Millipore |
| GSK3beta | P49841 | 9315 | Cell Signaling |
| GSK3beta-pSer9 | P49841 | 9336 | Cell Signaling |
| HDAC1 | Q13547 | 2062 | Cell Signaling |
| Her2 | P04626 | A0485 | Dako |
| HIF1beta(ARNT) | P27540 | 5537 | Cell Signaling |
| HistoneH3 | P68431 | 9715 | Cell Signaling |
| HLA-A,B,C | n.a. |  | AG Stevanovic |
| IDH1 | O75874 | 8137 | Cell Signaling |
| IGF1Rbeta | P08069 | 3018 | Cell Signaling |
| IGF1R-pTyr1135/Tyr1136 | P08069 | 3024 | Cell Signaling |
| IKKalpha | O15111 | 2682 | Cell Signaling |
| IKKalpha-pThr23 | O15111 | ab38515 | Abcam |
| IKKepsilon | Q14164 | 2905 | Cell Signaling |
| IKKepsilon-pSer172 | Q14164 | 8766 | Cell Signaling |
| JNK/SAPK | P45983 | 9252 | Cell Signaling |
| JNK/SAPK-pThr183/Tyr185 | P45983 | 4668 | Cell Signaling |
| Ki-67 | P46013 | K1700-05D | US Biologicals |
| LAMC1 | P11047 | 92921 | Cell Signaling |
| LDHA | P00338 | 2012 | Cell Signaling |
| MAD2L1 | Q13257 | 4636 | Cell Signaling |
| MEK1 | Q02750 | 9124 | Cell Signaling |
| MEK1/2-pSer217/221 | Q02750 | 9154 | Cell Signaling |
| MEK2 | P36507 | 9125 | Cell Signaling |
| mTOR(FRAP) | P42345 | 2983 | Cell Signaling |
| NF1(Neurofibromin) | P21359 | 14623 | Cell Signaling |
| NF-κBp65-pSer468 | Q04206 | 3039 | Cell Signaling |
| p38MAPK | Q16539 | 9212 | Cell Signaling |
| p38MAPK-pThr180/Tyr182 | Q16539 | 4511 | Cell Signaling |
| p53 | P04637 | 9282 | Cell Signaling |
| p53-pSer37 | P04637 | 9289 | Cell Signaling |
| p70S6kinase | P23443 | 2708 | Cell Signaling |
| p70S6kinase-pThr389 | P23443 | 9206 | Cell Signaling |
| PADI4 | Q9UM07 | sc-365369 | Santa Cruz |
| PAK1/2/3 | Q13153 | 2604 | Cell Signaling |
| PARP | P09874 | 9532 | Cell Signaling |
| PARP-cleavedAsp214 | P09874 | 9541 | Cell Signaling |
| PD1 | Q15116 | 86163 | Cell Signaling |
| PDK1 | Q15118 | 3062 | Cell Signaling |
| PDK1-pSer241 | Q15118 | 3061 | Cell Signaling |
| PD-L1 | Q9NZQ7 | 13684 | Cell Signaling |
| PI3-kinase p85 | P27986 | 4292 | Cell Signaling |
| PP2AC | P67775 | 2259 | Cell Signaling |
| PP2AC-pTyr307 | P67775 | AF3989 | R&D |
| PgR | P06401 | 8757 | Cell Signaling |
| PTEN | P60484 | 9552 | Cell Signaling |
| PTEN non-pSer380/Thr382/Thr383 | P60484 | 7960 | Cell Signaling |
| PTEN-pSer380 | P60484 | 9551 | Cell Signaling |
| Rad51 | Q06609 | ab109107 | Abcam |
| Ras | P01116 | 8955 | Cell Signaling |
| Rb-pSer795 | P06400 | 9301 | Cell Signaling |
| Rb-pSer807/811 | P06400 | 8516 | Cell Signaling |
| RSK1(p90RSK)-pThr573 | Q15418 | ab62324 | Abcam |
| S6RP | P62753 | 2217 | Cell Signaling |
| S6RP-pSer235/236 | P62753 | 2211 | Cell Signaling |
| Snail | O95863 | 3879 | Cell Signaling |
| Src | P12931 | 2109 | Cell Signaling |
| Src-pSer17 | P12931 | 5473 | Cell Signaling |
| Src-pTyr527 | P12931 | 2105 | Cell Signaling |
| STAT3 | P40763 | 4904 | Cell Signaling |
| STAT3-pTyr705 | P40763 | 9145 | Cell Signaling |
| Tau | P10636 | sc-32274 | Santa Cruz |
| Tau-pSer202 | P10636 | 39357 | Cell Signaling |
| Tubulin acetylated | P68366 | T6793 | Cell Signaling |
| Tubulinbeta-1chain | Q9H4B7 | MAB16676 | Abnova |
| Vimentin | P08670 | 5741 | Cell Signaling |
| Vimentin-pSer56 | P08670 | 7391 | Cell Signaling |

**
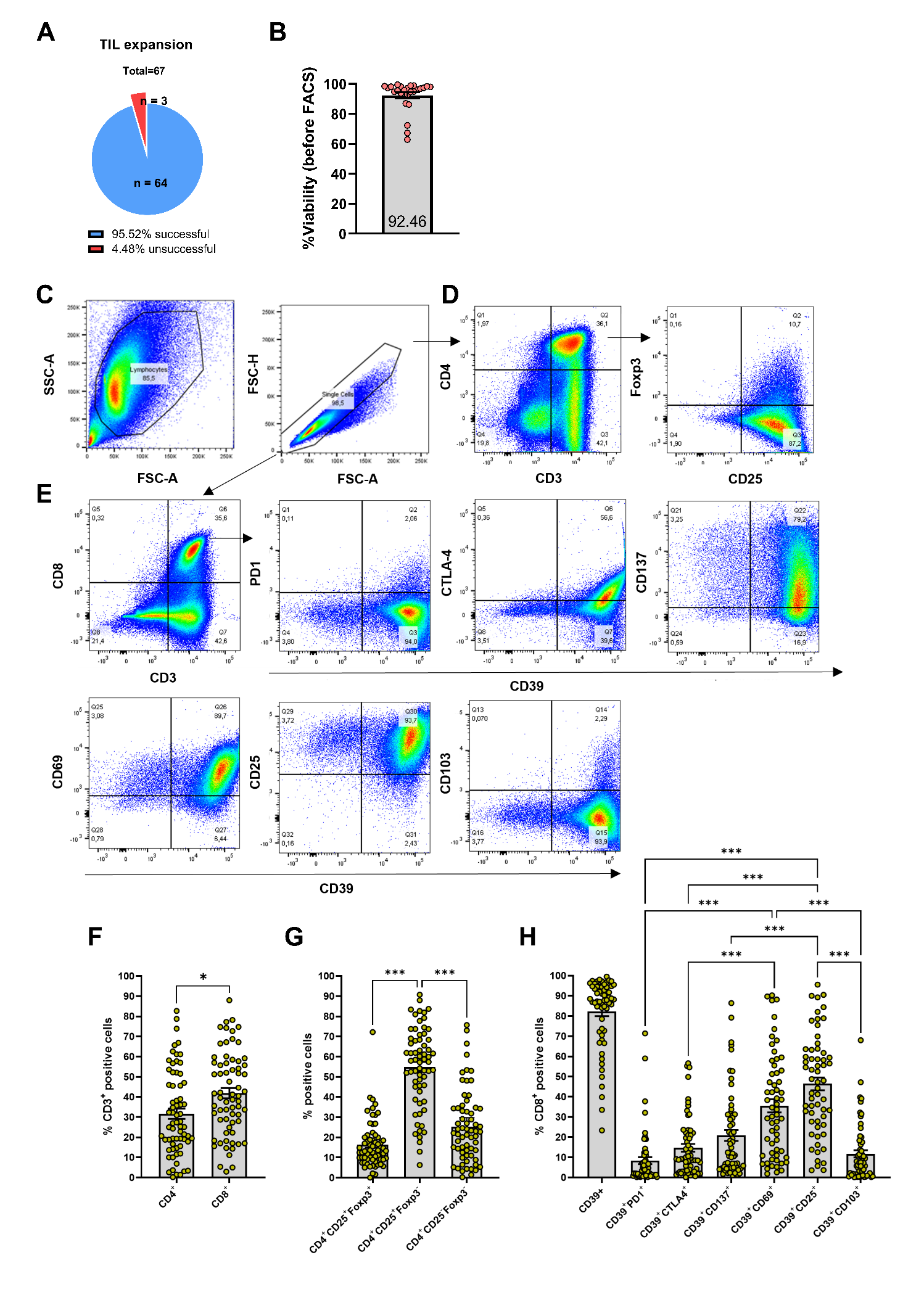
Supplementary Figures**

**
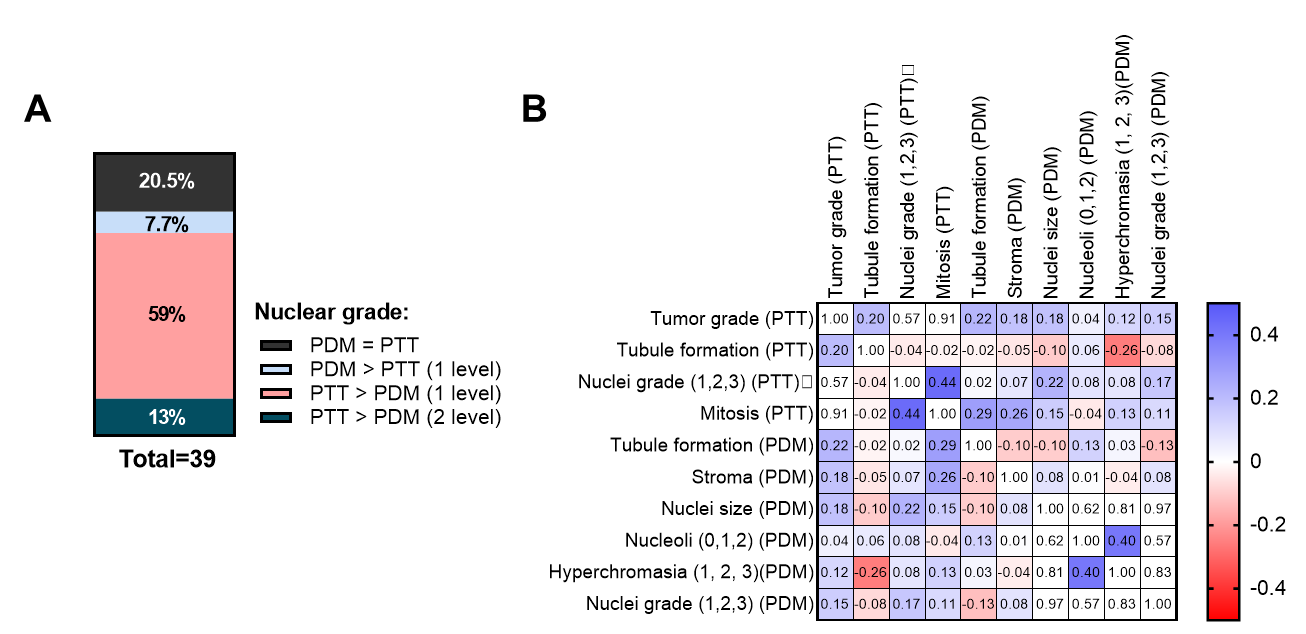
**

Figure S2. Correlation of nuclear grade in PDM and PTT. Nuclear grading of n = 39 PDM and PTT was assessed by a pathologist. In 20.5% of the specimen, nuclear grading of PTT was reflected by PDM. In 59% of the cases, PDM resembled a one-degree lower nuclear grade.

Figure S1. Multicolor flow cytometry analysis of isolated and expanded TILs from BC specimen. TILs were isolated in parallel to PDM isolation as a filtered single cell fraction from digested tumor samples. (A) TILs derived from n = 67 tissue samples were subsequently cultured, expanded and characterized by flow cytometry. BC-TIL expansion was highly successful with a 95.52% success rate (B) Viability of expanded TILs measured by NucleoCounter® NC-200™ prior to FACS staining. Average viability was 92.46%. (C) Gating strategy for lymphocyte and single cells. (D) Lymphocytes gated for CD3^+^CD4^+^ and regulatory T cells. (E) Lymphocytes gated for CD3^+^CD8^+^ cells. Cells are further differentiated into tumor-specific CD39^+^ cells expressing activation markers (PD1, CLTA-4, CD137, CD69, CD25) or the tissue-residence marker (CD103). (F-H) Cell frequencies of n = 67, n = 65 and n = 60 expanded BC-TIL samples; Paired t-test or Friedman test with Dunn’s multiple comparison test; *p < 0.05, **p < 0.01, ***p < 0.001. Data are mean with SEM.

**
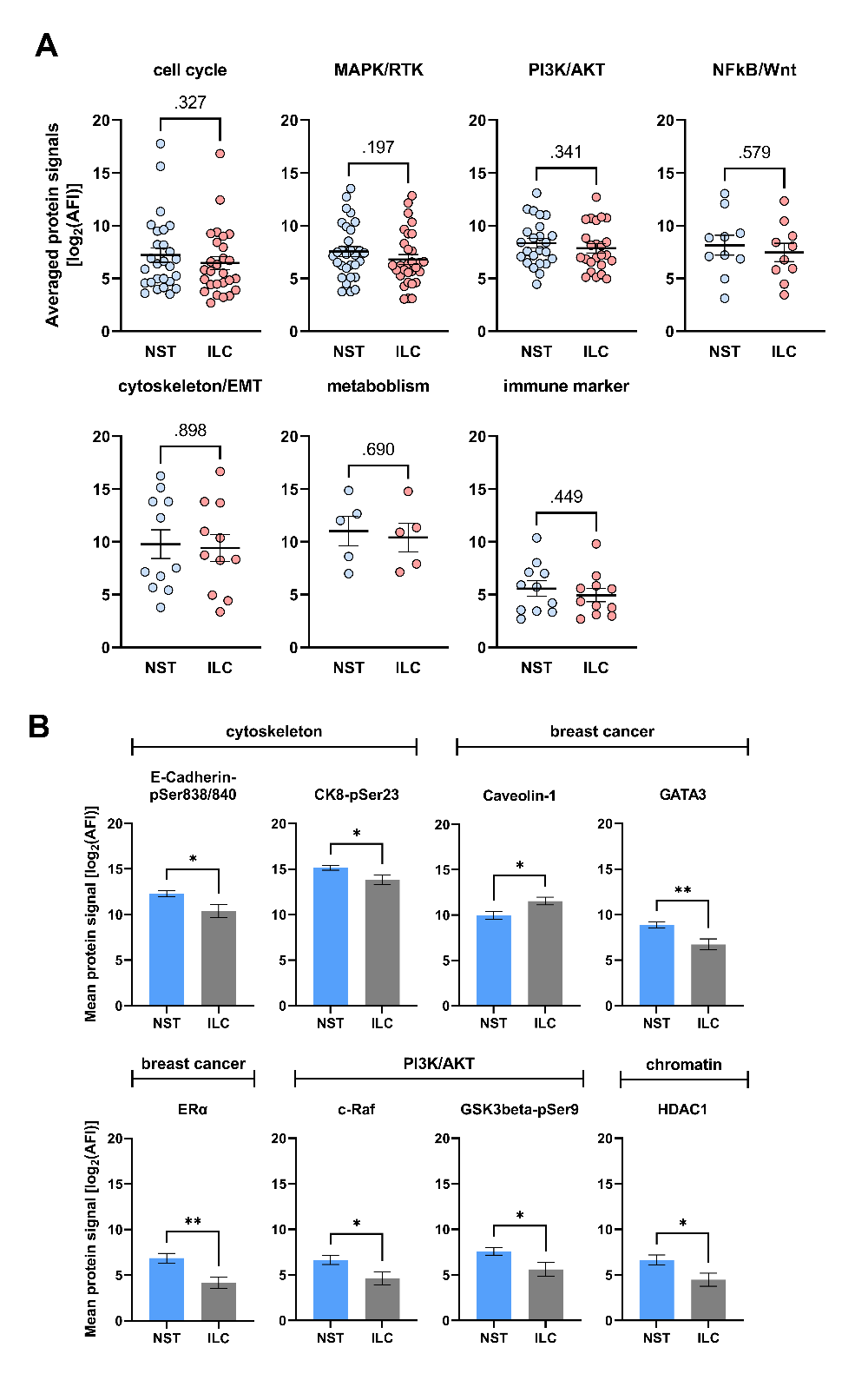
**

Figure S3. Proteomic comparison of NST and ILC-derived BC PDM. (A) Signaling pathway comparison of NST- and ILC-derived BC PDM. Normalized protein signals (AFI) in NST-/ILC-BC PDM were averaged and log2 transformed. No significant differences were detected. Mann-Whitney U test, p < 0.05.(B) Differentially expressed proteins in NST-/ILC-BC PDM. Enhanced protein abundances in NST- BC PDM were identified for several proteins associated with cell cytoskeleton, PI3K/AKT pathway and chromatin regulation, and with general breast cancer markers. Mann-Whitney U test, *p < 0.05, **p < 0.01, ***p < 0.001. Data are mean with SEM.

**
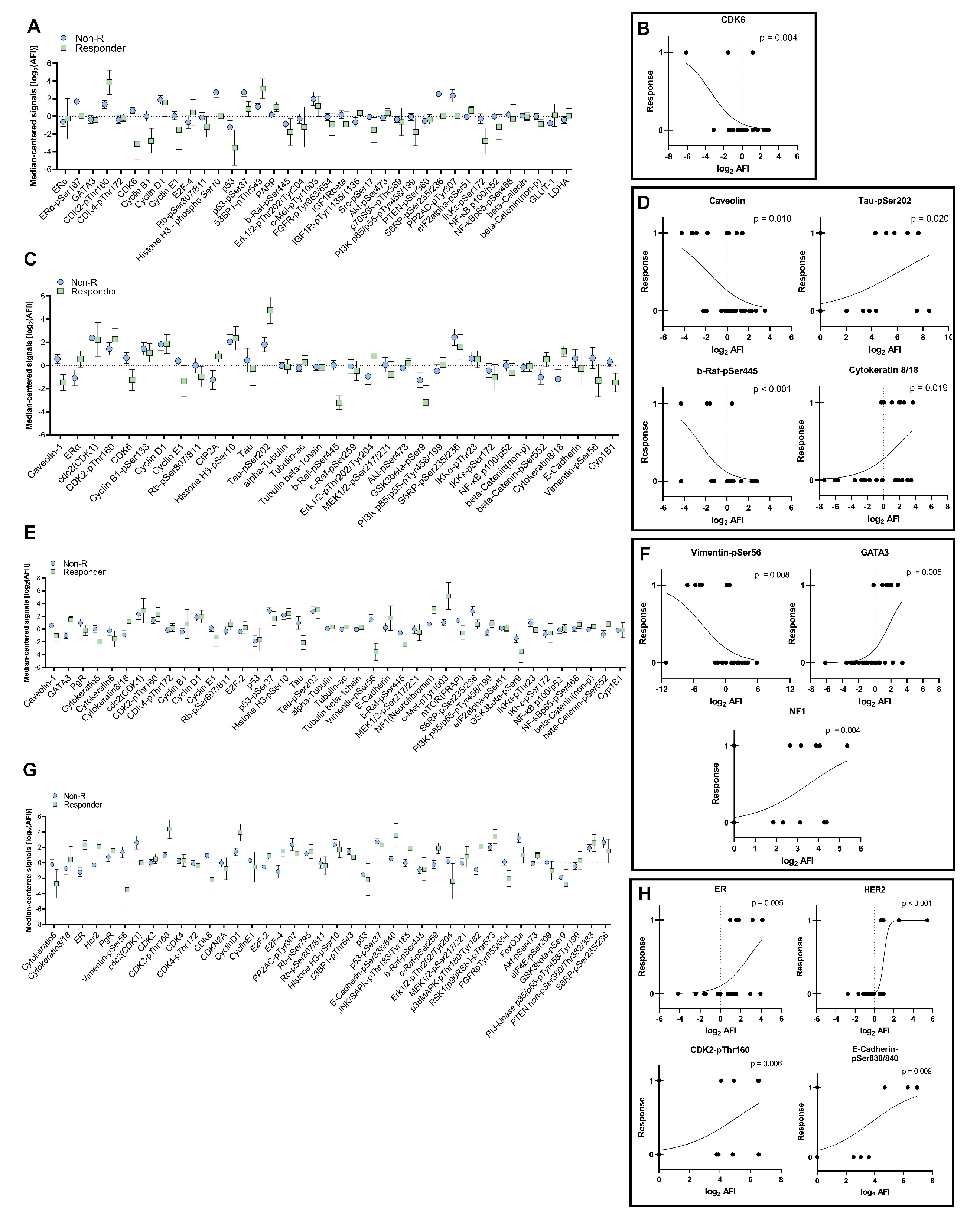
**

Figure S4. Identification of resistance and sensitivity marker panels in treatment responder and non-responder microtumors and regression analysis of differently expressed proteins. Microtumors were treated with four anti-cancer drugs and grouped into treatment responder and non-responders based on the results of cell death assays (CellTox ™ green cytotoxicity assay, Promega). DigiWest AFI protein signals of microtumors were median-centered, log2-transformed and compared between R and Non-R groups. Proteins that are associated with response/resistance to therapy according to literature, are differentially expressed, or are involved in therapy-related signaling pathways were plotted as interleaved scatter plots for tamoxifen (A), docetaxel (C), paclitaxel (E), and palbociclib (G) treatment. Blue symbols indicate the mean protein signal of the non-responder group, green symbols indicate the mean protein signal of the responder group. Simple logistic regression of differentially expressed proteins are depicted in (B) for tamoxifen, (D) for docetaxel, (F) for paclitaxel and (H) for palbociclib responses (1 = response; 0 = no response). Protein signals are displayed as log_2_ transformed AFI signals. As indicated by p-values (LRT) < 0.05, the amount of the shown proteins (predictor variables) significantly affected the likelihood of response to treatment. Descriptive statistics of simple logistic regressions are shown in Table S10. *p < 0.05, **p < 0.01 and ***p < 0.001. Shown are mean with SEM. Non-R and R. AFI: average fluorescent intensities; Non-R: non-responder; R: responder.

**Supplementary Tables**

**
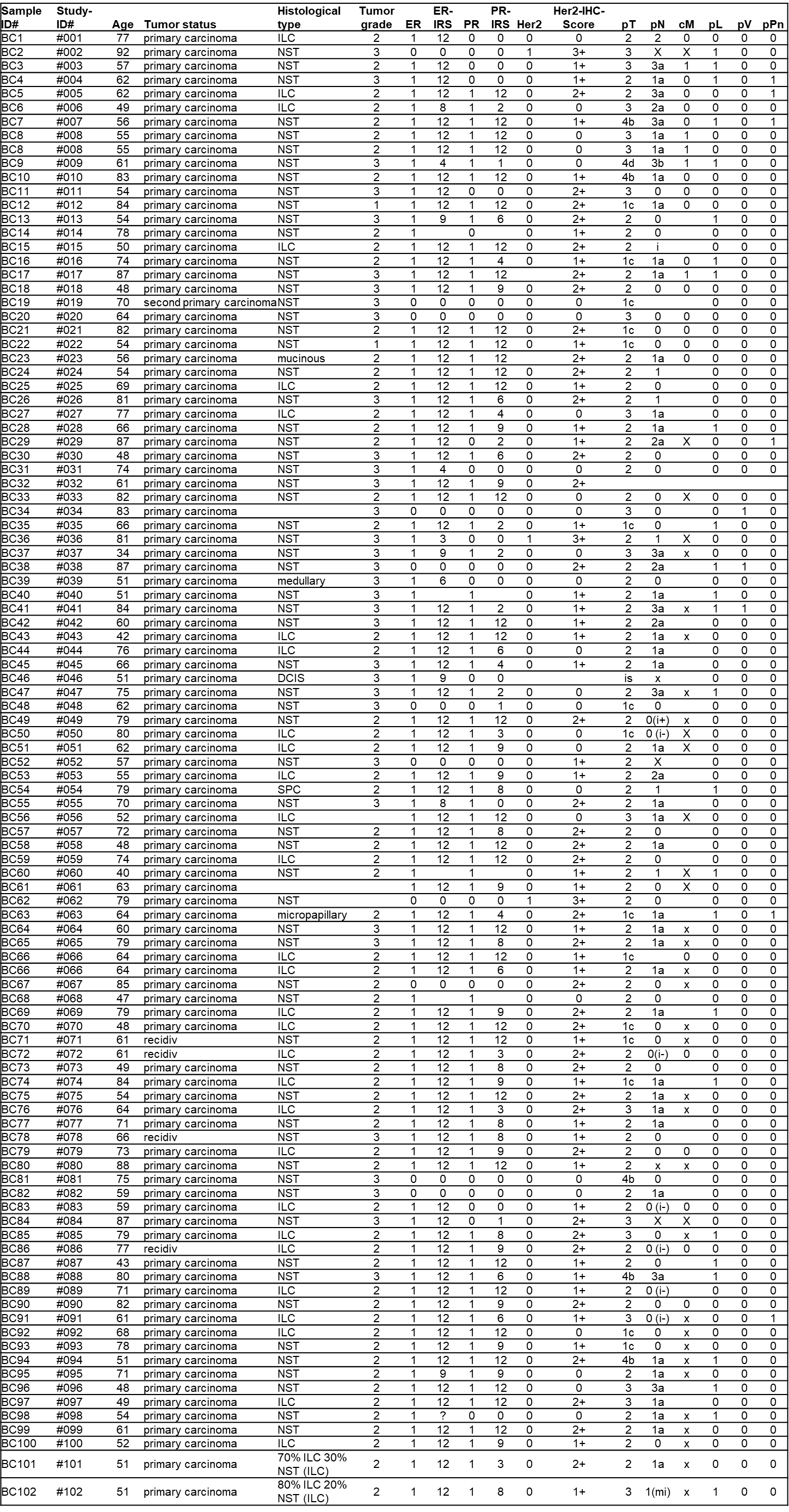
**

Table S1. Clinical patient data of the patient cohort.

**BC**: breast carcinoma; **NST**: invasive ductal carcinoma of no special type; **ILC**: invasive lobular carcinoma; **DCIS**: ductal carcinoma in-situ; **ER**: estrogen receptor status; **PR**: progesterone receptor status; **HER2**: HER2/neu-Erb-b2 receptor status; **IRS**: immunoreactive score (0-12); **p**: pathological; **c**: clinical; **T**: tumor size; **N**: lymph node spread; **M**: metastasis; **L**: lymphatic vessel spread; **V**: blood vessel spread; **Pn**: perineural invasion; “**0**” (ER, PR, HER2) = negative; “**1**” (ER, PR, HER2) = positive


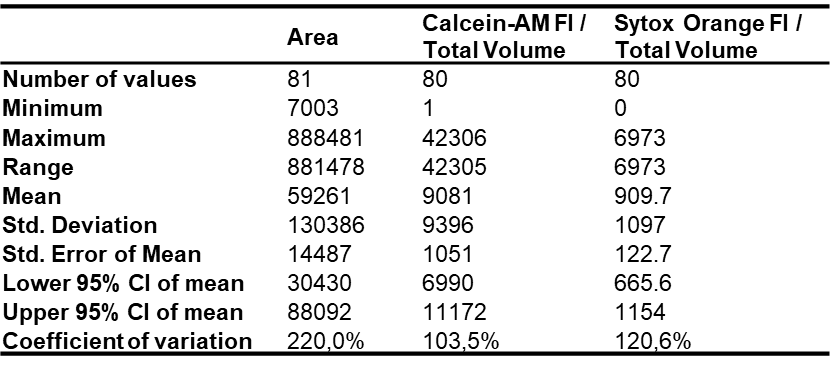
Table S2. Descriptive statistics of “area” and “fluorescent intensity live/dead” measurements in PDM.

Table S3. Raw data of DigiWest® protein signals in PDM and PTT samples and total measured protein amounts.

Table S4. DigiWest®-based AFI protein signals in matched PDM-PTT pairs DigiWest®-based AFI protein signals in matched PDM-PTT pairs.

Table S5. DigiWest®-based AFI protein signals of matched PDM-PTT pairs sorted by pathway affiliation


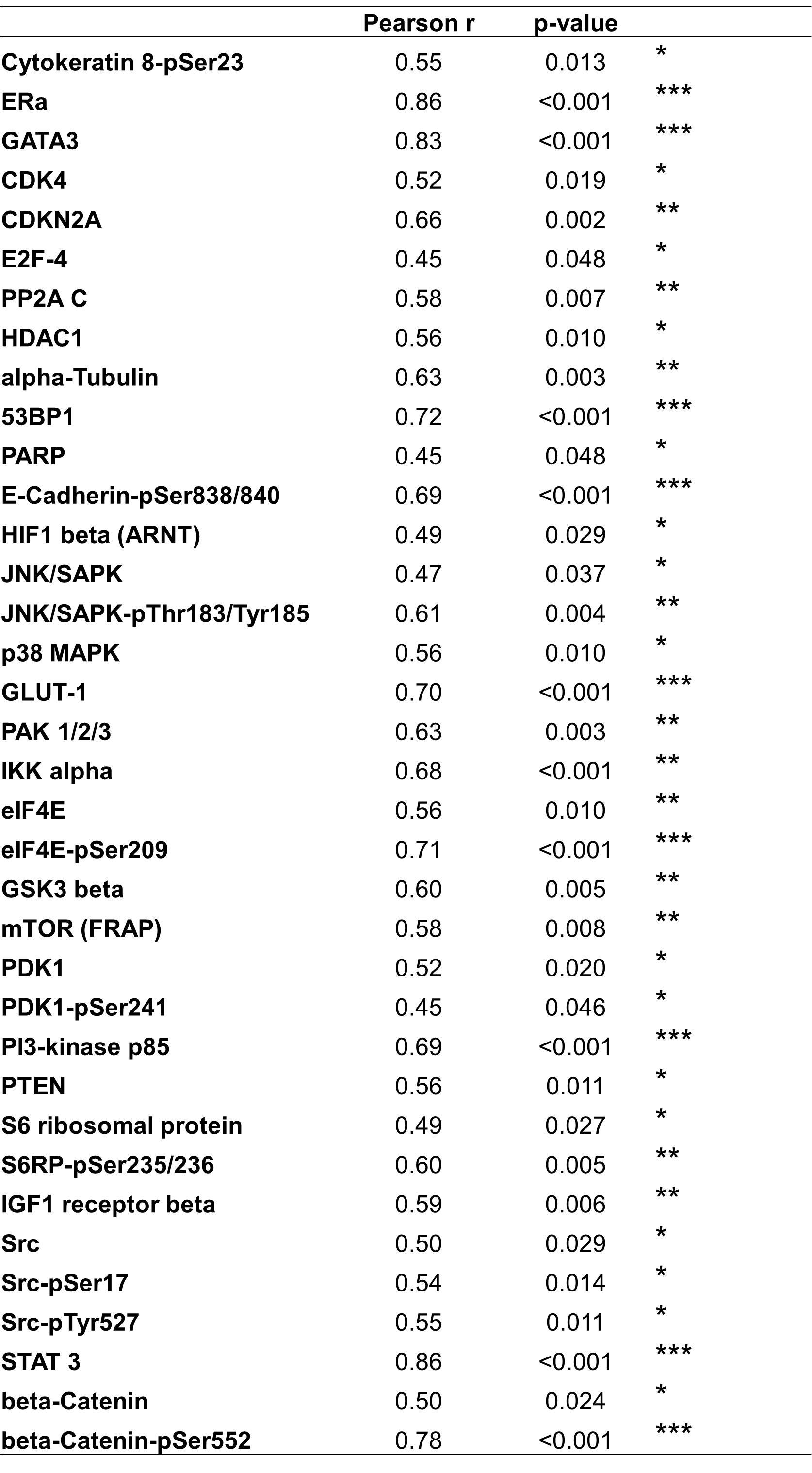


Table S6. Pearson correlation of protein abundances in PDM and corresponding PTT (PDM/PTT pairs).

Table S7. DigIWest®-based AFI protein signals of n = 42 PDM samples.


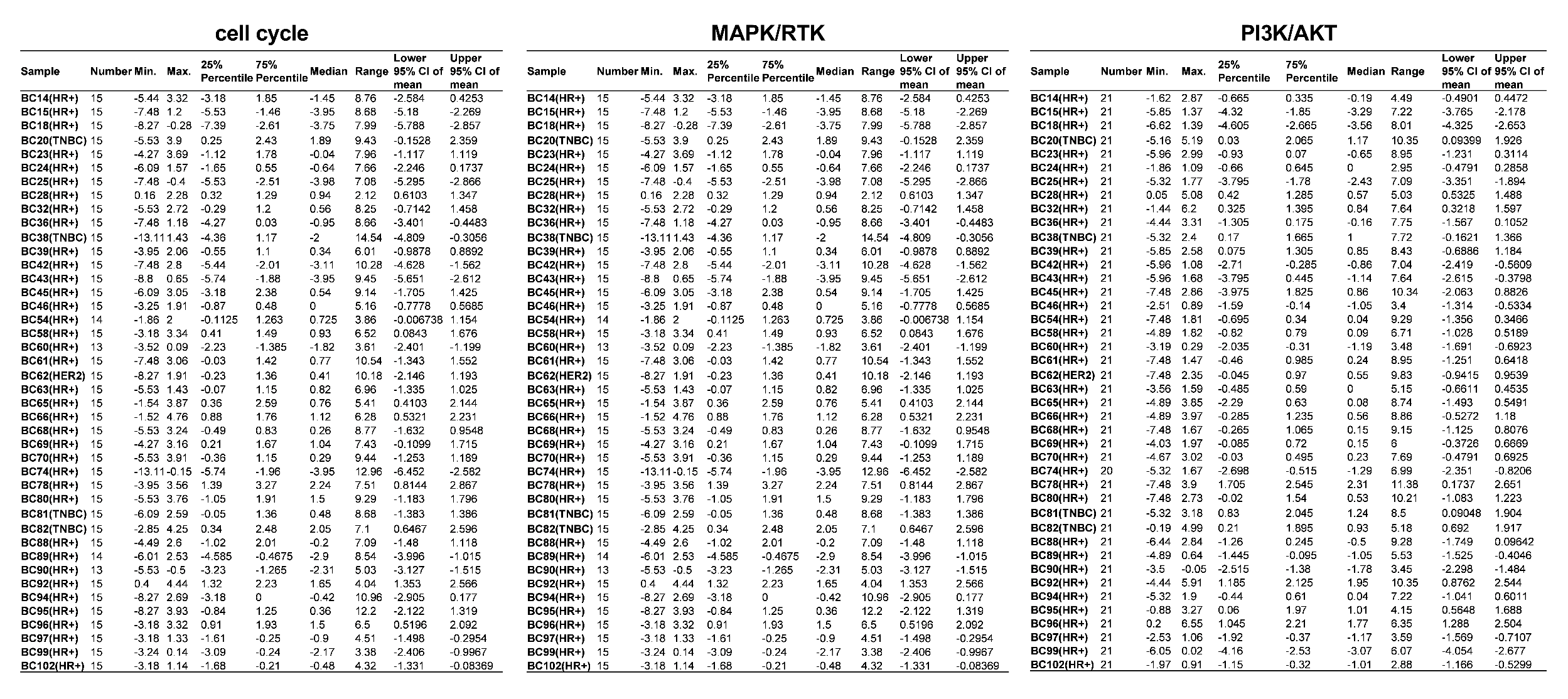


Table S9. Celltox™ Green assay RFU (relative fluorescent unit) values of BC microtumors treated with TAM, DTX, PTX and PAB.

Table S8. Descriptive statistics of averaged, median-centered and log2 transformed protein signals for cell cycle, MAPK/RTK and PI3K/AKT pathway in n = 42 PDM samples.


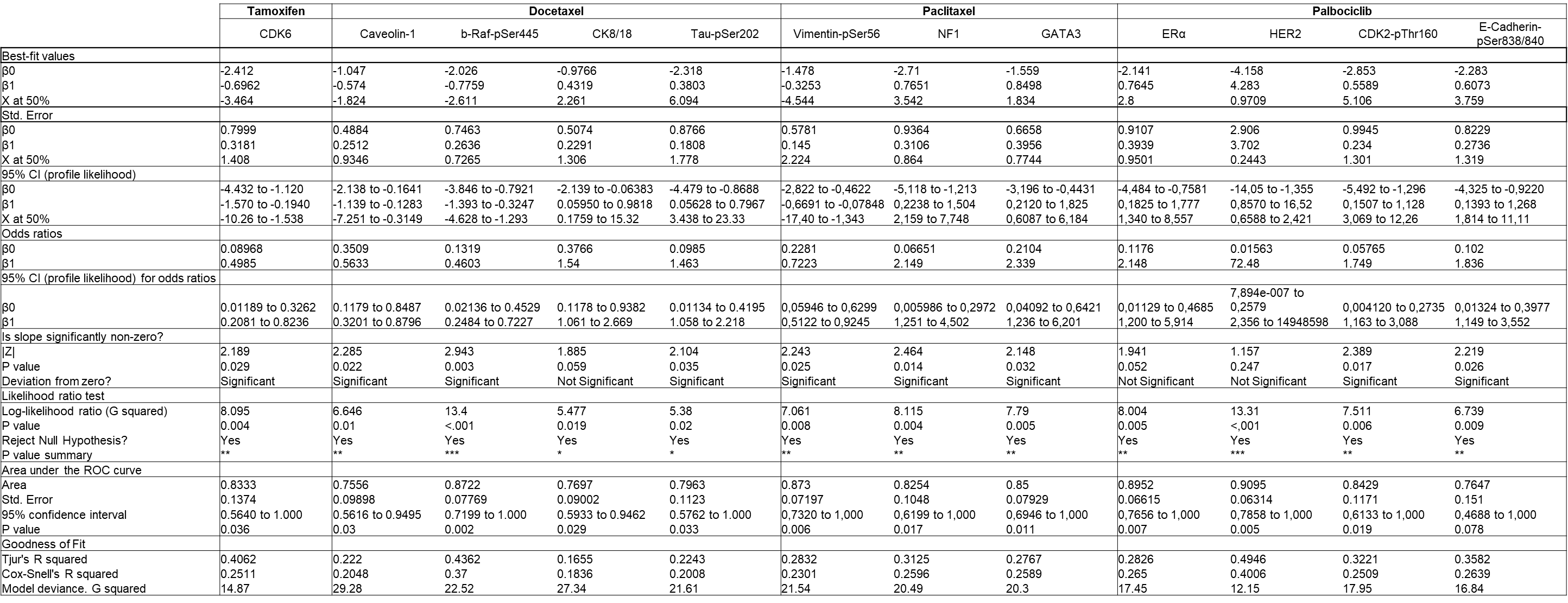


Table S10. Descriptive statistics of simple logistic regression analysis of differentially expressed proteins in treatment responder and non-responder groups.
